# Assembly drives regioselective azide-alkyne cycloaddition reaction

**DOI:** 10.1038/s41467-023-39658-0

**Published:** 2023-07-04

**Authors:** Qiaochu Jiang, Wenjun Zhan, Xiaoyang Liu, Lin Bai, Manli Wang, Ying Xu, Gaolin Liang

**Affiliations:** State Key Laboratory of Digital Medical Engineering, School of Biological Science and Medical Engineering, Southeast University, 2 Sipailou, Nanjing, 210096 China

**Keywords:** Organic chemistry, Self-assembly, Organocatalysis

## Abstract

Azide-alkyne cycloaddition reaction is a very common organic reaction to synthesize nitrogen-containing heterocycles. Once catalyzed by Cu(I) or Ru(II), it turns out to be a click reaction and thus is widely applied in chemical biology for labeling. However, besides their poor regioselectivity towards this reaction, these metal ions are not biologically friendly. Hence, it is an urgent need to develop a metal-free azide–alkyne cycloaddition reaction for biomedical applications. In this work, we found that, in the absence of metal ions, supramolecular self-assembly in an aqueous solution could realize this reaction with excellent regioselectivity. Nap-Phe-Phe-Lys(azido)-OH firstly self-assembled into nanofibers. Then, Nap-Phe-Phe-Gly(alkynyl)-OH at equivalent concentration approached to react with the assembly to yield the cycloaddition product Nap-Phe-Phe-Lys(triazole)-Gly-Phe-Phe-Nap to form nanoribbons. Due to space confinement effect, the product was obtained with excellent regioselectivity. Employing the excellent properties of supramolecular self-assembly, we are applying this strategy to realize more reactions without metal ion catalysis.

## Abstract (web summary)

Metal-free versions of azide–alkyne cycloadditions could find widespread applications in biomedical contexts. Here, the authors report an assembly-driven, regioselective azide–alkyne cycloaddition.

## Introduction

Cycloaddition reaction is one type of bimolecular reaction in which two conjugated reactants react to yield cyclic products^1^. As a typical cycloaddition reaction, azide–alkyne reaction has been extensively investigated and utilized. However, cycloadditions involving azides are limited by their reaction speeds (impractically slow at ambient temperature) and low regioselectivities of the products. Although high temperature can speed up the reaction, the mixtures of 1,4- and 1,5-disubstituted triazoles are formed in the reaction when the reactants are alkynes^2^. Therefore, many catalysts including copper(I) and ruthenium(II) have been exploited to quicken the reaction and improve product selectivity^3^. However, these metal ions are not biologically friendly, which limits further applications of the azide–alkyne cycloaddition reaction in vivo. Thus, suitable conditions for this reaction which can yield regionally selective products in the absence of metal ions at room temperature have been continuously explored, and some progresses have been made (e.g., photocatalysis and strain-promoted cycloadditions)^4–6^.

Self-assembly is a spontaneous process driven by thermodynamic and kinetic^7^. It is achieved (or influenced) by the synergistic effect of various non-covalent interactions, such as hydrogen bonding, π-π stacking, electrostatics, hydrophobicity, and van der Waals force^8^. The thermodynamic stability and the state of minimum energy of the ultimately formed nanostructures are determined by the synergistic effect of these non-covalent interactions^9,10^. Under the non-covalent interactions, small building blocks spontaneously and hierarchically assemble into functional supramolecular materials^11,12^. For example, artificial enzyme formed by self-assembly can function as mimic peroxidase^13^. Sureshan and coworkers reported topochemical azide–alkyne cycloaddition reaction using assembly of several peptide and sugar derivatives in crystal state^14^. However, crystallization often has some limitations such as low yield or unsuitability for large-scale synthesis. Alternatively, they utilized supramolecular self-assembly (i.e., hydrogelation) to achieve metal-free azide–alkyne cycloaddition under heating at high temperature^15,16^. Thus, directly and exclusively applying supramolecular self-assembly or its non-covalent reactions to realize a specific selective chemical reaction remains a great challenge. Specifically, using supramolecular self-assembly to drive metal-free azide–alkyne cycloaddition reaction without the assistance of any other beneficial factors (e.g., heating) has not been reported.

**Fig. 1:**
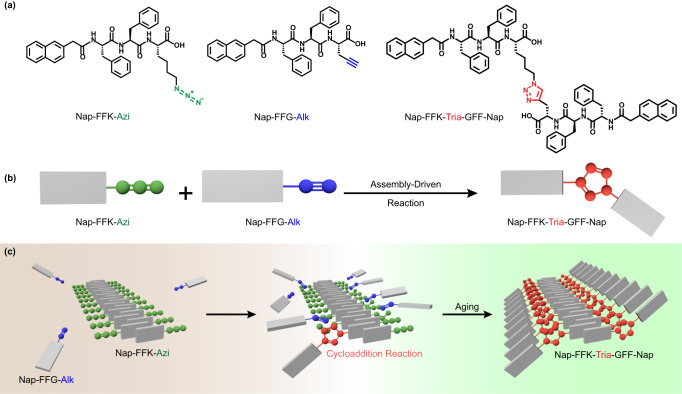
Regionally selective azide–alkyne cycloaddition reaction driven by assembly. **a** Chemical structures of **Nap-FFK-Azi,** **Nap-FFG-Alk** and **Nap-FFK-Tria-GFF-Nap**. **b** Diagram of the regionally selective azide–alkyne cycloaddition reaction driven by assembly. **c** Schematic illustration of the regionally selective azide–alkyne cycloaddition reaction driven by assembly.

Based on above-mentioned literature research, we are motivated to employ a typical supramolecular self-assembly system to realize a metal-free azide–alkyne cycloaddition reaction. Thus, we designed the self-assembling monomers Nap-Phe-Phe-Lys(azido)-OH (**Nap-FFK-Azi**) and Nap-Phe-Phe-Gly(alkynyl)-OH (**Nap-FFG-Alk**), as well as their cycloaddition product Nap-Phe-Phe-Lys(triazole)-Gly-Phe-Phe-Nap (**Nap-FFK-Tria-GFF-Nap**) (Fig. 1a). We speculate that, in aqueous solution, owing to the assembling ability of Nap-Phe-Phe (Nap-FF) moiety, **Nap-FFK-Azi** or **Nap-FFG-Alk** could spontaneously self-assemble into ordered nanostructures (always nanofibers). Along with the assembling process, **Nap-FFK-Azi** or **Nap-FFG-Alk** is drawn near to the nanofiber by the non-covalent interactions, driving the cycloaddition reaction to yield **Nap-FFK-Tria-GFF-Nap** (Fig. 1b). Hypothesis of the assembly-driven cycloaddition reaction process is summarized in Fig. 1c. Firstly, in the presence of two self-assembling monomers **Nap-FFK-Azi** and **Nap-FFG-Alk**, slim nanofibers are initiatively formed among the irregular aggregates through non-covalent interactions. Secondly, the distance between **Nap-FFK-Azi** and **Nap-FFG-Alk** gradually narrows, allowing effective molecule collision to realize azide–alkyne cycloaddition. Thirdly, accumulated product **Nap-FFK-Tria-GFF-Nap** transforms the slim nanofibers into wider ones (nanoribbons in this work).

## Results

### Syntheses and characterizations of hydrogels

**Fig. 2:**
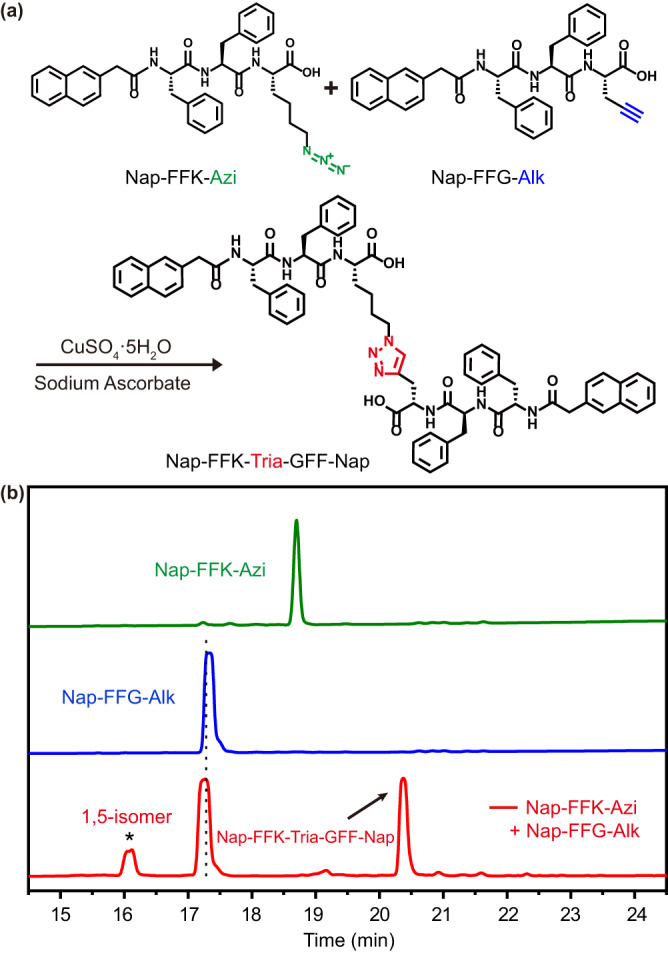
Cooper-catalyzed azide–alkyne cycloaddition (CuAAC) between Nap-FFK-Azi and Nap-FFG-Alk. **a** Synthetic route for **Nap-FFK-Tria-GFF-Nap**. Conditions: 20 mM **Nap-FFK-Azi**, 20 mM **Nap-FFG-Alk**, N,N-dimethylformamide (DMF), copper(I), 25 °C, and 6 h. **b** HPLC traces of **Nap-FFK-Azi** (green), **Nap-FFG-Alk** (blue), and the CuAAC reaction mixture in a (red). Wavelength for detection: 254 nm.

We first synthesized the three compounds **Nap-FFK-Azi,** **Nap-FFG-Alk**, and **Nap-FFK-Tria-GFF-Nap**. **Nap-FFK-Azi** and **Nap-FFG-Alk** were synthesized with solid phase peptide synthesis (SPPS), purified with high-performance liquid chromatography (HPLC), and then characterized with ESI-MS and ^1^H-NMR and ^13^C-NMR spectra (Supplementary Figs. 1–8). **Nap-FFK-Tria-GFF-Nap** was obtained from copper-catalyzed azide–alkyne cycloaddition (CuAAC) reaction (Fig. 2a). During the reaction, the mixture was injected into a HPLC system for analysis (Supplementary Table 1). As Fig. 2b shows, besides the desired product of **Nap-FFK-Tria-GFF-Nap** (retention time 20.3 min, 73.8%, Supplementary Figs. 9–11), the by-product 1,5-isomer of **Nap-FFK-Tria-GFF-Nap** (retention time 16.1 min, 26.2%, Supplementary Table 2, Supplementary Figs. 12 and 13) also appeared on the HPLC trace, suggesting poor regioselectivity of the copper-catalyzed azide–alkyne cycloaddition reaction.

To check the self-assembling ability of the three compounds, we measured their critical aggregation concentrations (CACs) in phosphate-buffered saline (PBS, pH 9). We chose this condition because these oligopeptides are hardly dissolved under pH 9. As shown in Supplementary Fig. 14, the CACs of **Nap-FFK-Azi,** **Nap-FFG-Alk**, and **Nap-FFK-Tria-GFF-Nap** were measured to be 40.6 μM, 263.8 μM, and 106.8 μM, respectively, suggesting **Nap-FFK-Azi** has the highest self-assembling ability among the three compounds. As we know, in aqueous solution, supramolecular hydrogelator containing Nap-FF self-assembling moiety always self-assembles into nanofibers to form macroscopic hydrogel^17,18^. In this work, we found that **Nap-FFK-Azi** and **Nap-FFG-Alk** hydrogels could be obtained via a heating-cooling method. As we mentioned above, high temperature can speed up the azide–alkyne cycloaddition reaction. Thus, to avoid the occurrence of the cycloaddition reaction between **Nap-FFK-Azi** and **Nap-FFG-Alk** while in the meantime assure the occurrence of their hydrogelations, we heated these two compounds at 100 °C in PBS and used HPLC to trace their cycloaddition product (i.e., **Nap-FFK-Tria-GFF-Nap**) at different times. The results in Supplementary Fig. 15 shows that 1 h heating of the two compounds at 2.5 mM did not yield the product **Nap-FFK-Tria-GFF-Nap** while 2 h heating did. Note that these oligopeptides are hardly dissolved in aqueous solutions at concentrations higher than 2.5 mM under above heating conditions. Thus, a hydrogelator concentration of 2.5 mM, as well as 1 h heating at 100 °C was chosen as the condition for following hydrogelation experiments. After obtaining the **Nap-FFK-Azi** and **Nap-FFG-Alk** hydrogels with heating-cooling method (heating at 100 °C for 1 h and cooling at 4 °C for 24 h, compound concentration: 2.5 mM), we used dynamic rheological measurements to evaluate their physical properties. As shown in Supplementary Figs. 16 and 17, the dynamic storage modulus (G′) of these two hydrogels were apparently higher than their corresponding loss modulus (G″), indicating both hydrogels can endure external shear force. However, upon the same heating-cooling treatment, 2.5 mM **Nap-FFK-Tria-GFF-Nap** remained a clear solution, indicating the compound might have self-assembled but could not form a hydrogel. Interestingly, when we applied above hydrogelation method on the mixture solution of **Nap-FFK-Azi** and **Nap-FFG-Alk** at 2.5 mM, a transparent hydrogel was obtained. And the **Nap-FFK-Azi** + **Nap-FFG-Alk** hydrogel had much higher G′ and G′′ values than above two hydrogels, respectively (Supplementary Fig. 18). Because the CAC value of **Nap-FFK-Azi** is much lower than that of **Nap-FFG-Alk**, we hypothesized that, in the **Nap-FFK-Azi** + **Nap-FFG-Alk** hydrogel, **Nap-FFK-Azi** precede **Nap-FFG-Alk** in self-assembling state. And HPLC analysis indicated that, at this hydrogelation condition, the cycloaddition product **Nap-FFK-Tria-GFF-Nap** was barely seen in the **Nap-FFK-Azi** + **Nap-FFG-Alk** hydrogel (Supplementary Fig. 19).

### Monitoring of assembly-driven azide–alkyne cycloaddition reaction

**Fig. 3:**
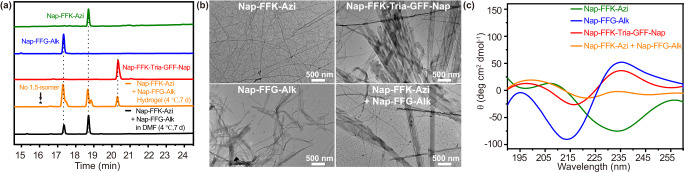
Characterizations of assembly-driven azide–alkyne cycloaddition reaction. **a** HPLC traces of 2.5 mM **Nap-FFK-Azi** (green), 2.5 mM **Nap-FFG-Alk** (blue), 2.5 mM **Nap-FFK-Tria-GFF-Nap** (red), 2.5 mM **Nap-FFK-Azi** + **Nap-FFG-Alk** hydrogel at 4 °C for 7 d (orange), and 2.5 mM **Nap-FFK-Azi** + **Nap-FFG-Alk** in DMF at 4 °C for 7 d (black). Wavelength for detection: 254 nm. **b** TEM images of 2.5 mM **Nap-FFK-Azi** hydrogel, 2.5 mM **Nap-FFG-Alk** hydrogel, 2.5 mM **Nap-FFK-Tria-GFF-Nap** solution, and 2.5 mM **Nap-FFK-Azi** + **Nap-FFG-Alk** hydrogel after aging at 4 °C for 7 d. scale bar = 500 nm. **c** CD spectra of the samples in (**b**).

To test our hypothesis of assembly-driven azide–alkyne cycloaddition, we used HPLC to analyze the chemical composition of the **Nap-FFK-Azi** + **Nap-FFG-Alk** hydrogel during its aging at 4 °C. As shown in Fig. 3a, after 7 d aging, desired 1,4-disubstituted cycloaddition product **Nap-FFK-Tria-GFF-Nap** (HPLC retention time 20.3 min, 14.7%, Supplementary Fig. 20 and Supplementary Table 3) was yielded in the hydrogel. Interestingly, the 1,5-isomer of **Nap-FFK-Tria-GFF-Nap**, which was also another product of the CuAAC between **Nap-FFK-Azi** and **Nap-FFG-Alk** (Fig. 2b), was not found in the hydrogel (Fig. 3a), suggesting excellent regioselectivity of this assembly-driven cycloaddition reaction. We noticed that two small shoulder peaks (17.5 min and 18.7 min) in the HPLC trace of **Nap-FFK-Azi** + **Nap-FFG-Alk** hydrogel (Fig. 3a). ESI-MS analyses indicated that they were the same substances as those at 17.3 min and 18.6 min, respectively (Supplementary Figs. 21 and 22). Above peak splitting may owe to the sample being prepared under alkaline conditions but tested in an acidic environment. The reaction was also monitored under different pH values ranging from 9 to 11 and concentrations ranging from 0.625 mM to 2.5 mM, and we found that pH variations affected the cycloaddition reaction very tiny while concentration did much. As shown in Supplementary Fig. 23, at pH 9–11, the reaction shared vicinal yields of 6.63–6.91%. In contrast, when the concentration was increased from 0.625 mM to 2.5 mM, the corresponding yield increased from 1.53% to 6.91% (Supplementary Figs. 23 and 24), indicating that the assembly-driven azide–alkyne cycloaddition reaction is concentration-dependent. We further monitored the reaction in the hydrogel till 36 d using HPLC analysis. As displayed in Supplementary Fig. 25, the reaction yield increased gradually and reached its plateau of 19.1% at 14 d.

As we know, those necessary conditions for supramolecular self-assembly in aqueous solution (e.g., hydrophobic interaction, π-π stacking, hydrogen bonding, etc) do not exist in organic solvent such as *N*,*N*-dimethylformamide (DMF). Thus, to validate that above product **Nap-FFK-Tria-GFF-Nap** was yielded under assembly-induced space confinement effect, we incubated 2.5 mM **Nap-FFK-Azi** + **Nap-FFG-Alk** in DMF at 4 °C for 7 d and injected the mixture for HPLC analysis. Clearly, when **Nap-FFK-Azi** and **Nap-FFG-Alk** remained monomeric state in DMF, no cycloaddition product of **Nap-FFK-Tria-GFF-Nap** was found in the incubation (Fig. 3a), indicating that the product in above **Nap-FFK-Azi** + **Nap-FFG-Alk** hydrogel was indeed yielded under assembly effect. In order to eliminate the possibility that the solvent of **Nap-FFK-Azi** + **Nap-FFG-Alk** hydrogel (i.e., PBS) could induce the cycloaddition reaction between **Nap-FFK-Azi** and **Nap-FFG-Alk**, we added 2.5 mM Lys(azido)-OH (**K-Azi**) and Gly(alkynyl)-OH (**G-Alk**) to PBS, and applied mixture solution to above heating-cooling procedure. Without the self-assembling motif Nap-FF, neither **K-Azi** nor **G-Alk** could self-assemble into nanofibers in PBS. As expected, we did not find any fibrous structure from the transmission electron microscopy (TEM) image of above PBS solution (Supplementary Fig. 26). HPLC analyses consistently showed that, up to 14 d aging at 4 °C, no cycloaddition product between **K-Azi** and **G-Alk** (i.e., Lys(traizole)Gly, **K-Tria-G**) was found in above mixture PBS solution (Supplementary Figs. 27–30). Above results collectively indicate that, it is assembly but not the solvent that drives the metal-free azide–alkyne cycloaddition reaction to yield the regionally selective product.

We further used TEM observations to validate above assemble-driven cycloaddition in **Nap-FFK-Azi** + **Nap-FFG-Alk** hydrogel. As shown in Fig. 3b, after 7 d aging, while dense and slim nanofibers were observed in the TEM image of **Nap-FFK-Azi** hydrogel (average diameter: ~20 nm), helical ribbons with an average diameter of 40 nm were found in the TEM image of **Nap-FFG-Alk** hydrogel. In the TEM of **Nap-FFK-Tria-GFF-Nap** solution after same treatment, helical ribbons with an average diameter of 110 nm presented. Interestingly, in the TEM image of **Nap-FFK-Azi** + **Nap-FFG-Alk** hydrogel after 7 d aging, besides slim nanofibers, helical ribbons with an average diameter around 130 nm were clearly observed (Fig. 3b). To rule out the possibility that co-assembly of the two precursor peptides also result in the final nanoribbons, a nonreactive control Nap-Phe-Phe-Nva-OH (**Nap-FF-Nva**, by replacing the alkyne group of **Nap-FFG-Alk** with an alkyl group) was synthesized for comparative study (Supplementary Figs. 31–33). Cycloaddition cannot take place between **Nap-FFK-Azi** and **Nap-FF-Nva** as expected (Supplementary Fig. 34), while the possibility of co-assembly among them remains. Upon the same heating-cooling treatment, both 2.5 mM **Nap-FF-Nva** and 2.5 mM **Nap-FFK-Azi** + **Nap-FF-Nva** could form stable and transparent hydrogels (Supplementary Figs. 35 and 36). As shown in Supplementary Fig. 37, at day 7, similar nanoribbons to those in **Nap-FFG-Alk** hydrogel with an average diameter of 125 nm were also observed in **Nap-FF-Nva** hydrogel. However, only slim nanofibers (average diameter: ~18 nm) but not nanoribbons were found in **Nap-FFK-Azi** + **Nap-FF-Nva** hydrogel. Above observations indicated that co-assembly of the peptides most likely lead to the formation of nanofibers rather than nanoribbons in **Nap-FFK-Azi** + **Nap-FF-Alk** hydrogel. Since the assemblies of **Nap-FFK-Tria-GFF-Nap** were significantly wider than those of **Nap-FFK-Azi** or **Nap-FFG-Alk**, from the TEM image of **Nap-FFK-Azi** + **Nap-FFG-Alk** hydrogel we knew that cycloaddition reaction between **Nap-FFK-Azi** and **Nap-FFG-Alk** indeed occurred in the hydrogel.

To further validate the nanoribbons in **Nap-FFK-Azi** + **Nap-FFG-Alk** hydrogel were in accordance with those in **Nap-FFK-Tria-GFF-Nap** solution, we used circular dichroism (CD) to investigate the secondary structures of the assemblies in above four samples. As displayed in Fig. 3c, CD spectrum of **Nap-FFK-Azi** hydrogel exhibited a negative peak at 197 nm and a positive peak at 207 nm, indicating its nanofibers adopt β-turn-like structures. In contrast, a positive peak at 195 nm and a negative peak at 215 nm appeared in the CD spectrum of **Nap-FFG-Alk** hydrogel, suggesting the helical nanoribbons in the hydrogel adopt β-sheet-like structures. A positive peak at 197 nm and a negative peak at 217 nm appeared in the CD spectrum of **Nap-FFK-Tria-GFF-Nap** solution, suggesting β-sheet-like structures of its nanoribbons. Interestingly, a positive peak at 199 nm and a negative peak at 222 nm, both of which are closer to those of **Nap-FFK-Tria-GFF-Nap** solution than to those of **Nap-FFG-Alk** hydrogel, appeared in the CD spectrum of **Nap-FFK-Azi** + **Nap-FFG-Alk** hydrogel, suggesting the nanoribbons in the hydrogel are made of **Nap-FFK-Tria-GFF-Nap**. We noted that the CD spectra of these four samples all showed positive/negative peaks at 235 nm, which should be assigned to the different conformation of their chiral phenylalanine residues^19^. The negative peak at 235 nm of **Nap-FFK-Azi** hydrogel was induced by its β-turn-like nanofibers, while the positive peaks at 235 nm in other three groups were induced by their corresponding β-sheet-like nanostructures. We also performed CD analyses of **Nap-FF-Nva** hydrogel and **Nap-FFK-Azi** + **Nap-FF-Nva** hydrogel. CD spectrum of **Nap-FF-Nva** hydrogel exhibited a positive peak at 199 nm and a negative peak at 218 nm, indicating a β-sheet-like structure of the nanoribbons in the hydrogel. This observation was consistent with that of the **Nap-FFG-Alk** hydrogel. For **Nap-FFK-Azi** + **Nap-FF-Nva** hydrogel, two negative peaks at 194 nm and 222 nm and a positive peak at 208 nm appeared in its CD spectrum (Supplementary Fig. 38), suggesting a complex structure rather than a single secondary structure in the hydrogel. We believe this complex CD spectrum is more likely to be a spectral superposition. In contrast, **Nap-FFK-Azi** + **Nap-FFG-Alk** hydrogel presented a distinct secondary structure that is similar to that of **Nap-FFK-Tria-GFF-Nap** assemblies (Fig. 3c). Taken together, these results additionally confirmed that cycloaddition reaction between **Nap-FFK-Azi** and **Nap-FFG-Alk** indeed occurred in **Nap-FFK-Azi** + **Nap-FFG-Alk** hydrogel.

### Mechanism of assembly-driven azide–alkyne cycloaddition reaction

**Fig. 4:**
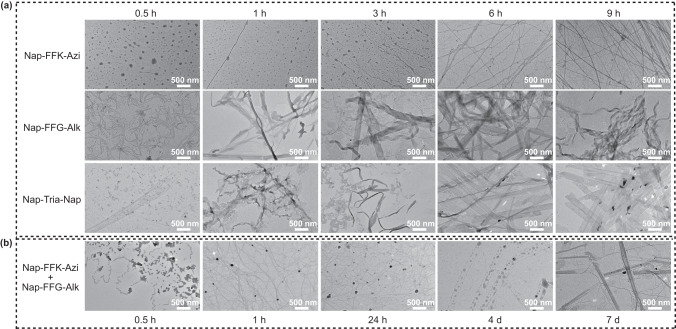
Real-time TEM observations of the assembly-driven regionally selective azide–alkyne cycloaddition reaction. **a** Real-time TEM images of 2.5 mM **Nap-FFK-Azi** hydrogel, 2.5 mM **Nap-FFG-Alk** hydrogel, and 2.5 mM **Nap-FFK-Tria-GFF-Nap** solution aging at 4 °C. **b** Real-time TEM images of 2.5 mM **Nap-FFK-Azi** + **Nap-FFG-Alk** hydrogel aging at 4 °C. scale bar = 500 nm.

To validate the mechanism of assembly-driven regionally selective azide–alkyne cycloaddition reaction shown in Fig. 1c, we used TEM observations to real-time monitor the formation of above nanostructures. TEM images in Fig. 4a show that, at 0.5 h, those raw materials at nanoscale for self-assembling into bigger nanostructures (e.g., nanoparticles for nanofiber formation, nanofibers for nanoribbon formation) started to appear in hydrogels of **Nap-FFK-Azi** and **Nap-FFG-Alk**, or solution of **Nap-Trai-Nap**. The final nanostructures (i.e., nanofibers in hydrogel **Nap-FFK-Azi**, nanoribbons in hydrogel **Nap-FFG-Alk** and solution **Nap-Trai-Nap**) started to form at 3 h and stabilize at 6 h. In contrast, TEM images of **Nap-FFK-Azi** + **Nap-FFG-Alk** hydrogel showed that dense and slim nanofibers (i.e., **Nap-FFK-Azi** nanofibers) formed at 3 h (Fig. 4b). After that, from 24 h to 4d, we found that nanoparticles (i.e., generation of the cycloaddition product **Nap-Trai-Nap**) were continuously generated along the nanofibers. Finally, the nanoparticles connected with each other to form helical nanoribbons at day 7. In contrast, the real-time formation of nanostructures in **Nap-FFK-Azi** + **Nap-FF-Nva** hydrogel displayed completely different assembly processes. As shown in Supplementary Fig. 39, an obvious nanoribbon-nanofiber transformation was observed in **Nap-FFK-Azi** + **Nap-FF-Nva** hydrogel. Compared with those in the reactive group (**Nap-FFK-Azi** + **Nap-FFG-Alk**), the wide nanoribbon structures in **Nap-FFK-Azi** + **Nap-FF-Nva** hydrogel formed merely by co-assembly were not stable and transformed into fine nanofibers at day 7, indicating they were sub-equilibrium intermediates during the co-assembly process. This result also indicates that occurrence of the cycloaddition reaction in **Nap-FFK-Azi** + **Nap-FFG-Alk** group improves the stability of the intermediate nanoribbon structure of the two precursor peptides to the final nanoribbons. In addition, different from that in **Nap-FFK-Azi** + **Nap-FFG-Alk** group, no nanoparticle structure was found in **Nap-FFK-Azi** + **Nap-FF-Nva** group from 24 h to 7d. Above TEM observations directly validate our hypothesis that, along with the self-assembly of nanofiber, **Nap-FFK-Azi** or **Nap-FFG-Alk** is drawn near to the nanofiber with space-confined effect, undergoing cycloaddition reaction to yield regionally selective product **Nap-FFK-Tria-GFF-Nap**.

### Influence of amino acid chirality on assembly-driven azide–alkyne cycloaddition reaction

**Fig. 5:**
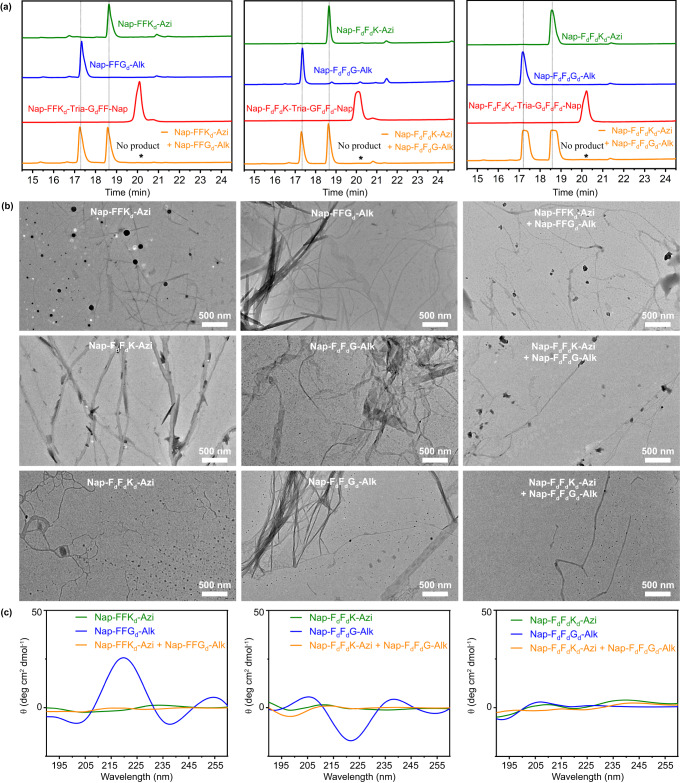
Characterizations of the assemblies of chiral peptides. **a** HPLC traces of 2.5 mM chiral isomers of **Nap-FFK-Azi** (green), 2.5 mM chiral **Nap-FFG-Alk** (blue), 2.5 mM chiral **Nap-FFK-Tria-GFF-Nap** (red), or 2.5 mM chiral **Nap-FFK-Azi + Nap-FFG-Alk** hydrogel (orange) at 4 °C for 7 d. Wavelength for detection: 254 nm. **b** TEM images of the chiral isomer **Nap-FFK-Azi** hydrogel, 2.5 mM chiral **Nap-FFG-Alk** hydrogel, or 2.5 mM chiral **Nap-FFK-Azi + Nap-FFG-Alk** hydrogel after aging at 4 °C for 7 d. scale bar = 500 nm. **c** CD spectra of the corresponding samples in (**b**).

In order to investigate the effect of amino acid chirality on this assembly-driven cycloaddition reaction, we designed and synthesized three sets of control compounds: (i) group C1: **Nap-FFK**_**d**_**-Azi,** **Nap-FFG**_**d**_**-Alk**, and **Nap-FFK**_**d**_**-Tria-G**_**d**_**FF-Nap**; (ii) group C2: **Nap-F**_**d**_**F**_**d**_**K-Azi,** **Nap-F**_**d**_**F**_**d**_**G-Alk**, and **Nap-F**_**d**_**F**_**d**_**K-Tria-GF**_**d**_**F**_**d**_**-Nap**; (iii) group C3: **Nap-F**_**d**_**F**_**d**_**K**_**d**_**-Azi,** **Nap-F**_**d**_**F**_**d**_**G**_**d**_**-Alk**, and **Nap-F**_**d**_**F**_**d**_**K**_**d**_**-Tria-G**_**d**_**F**_**d**_**F**_**d**_**-Nap**. All the compounds were synthesized and purified with similar protocols, and then characterized with ESI-MS, ^1^H-NMR and ^13^C-NMR spectra (Supplementary Figs. 40–60). CAC measurement showed that their CACs were much smaller than 2.5 mM (Supplementary Fig. 61), suggesting these are able to self-assemble under a heat-cooling process (Supplementary Figs. 62–66). However, none of the cycloaddition products (retention time 20.3 min) was monitored by HPLC in these chiral groups (Fig. 5a). Significantly shorter, fragile fibers with indistinct edges, either linear or banded, were observed in TEM images of the individual peptides (Fig. 5b). However, in the groups of mixed peptides (e.g., **Nap-FFK**_**d**_**-Azi** + **Nap-FFG**_**d**_**-Alk**), only a few fragile linear nanostructures were appeared in their TEM images while nanoribbons with larger size were not observed at all (Fig. 5b). These results suggested that, after the chirality of its amino acid was changed, self-assembling property of the peptide was greatly affected, and co-assembly between different peptides was almost impossible to occur. As a result, assembly-driven cycloaddition reaction was inhibited. This hypothesis was further validated by CD spectra of these peptide assemblies. As displayed in Fig. 5c, only the CD spectra of **Nap-FFG**_**d**_**-Alk** and **Nap-F**_**d**_**F**_**d**_**G-Alk** displayed obvious secondary structures, but they are not the typical structures of α-helix, β-turn, or β-sheet, indicating their assemblies were complex. In general, the change of chirality showed a significant inhibitory effect on the assembly process, as well as the assembly-driven azide–alkyne cycloaddition reaction.

## Discussion

In summary, we rationally designed three compound (**Nap-FFK-Azi,** **Nap-FFG-Alk**, and **Nap-FFK-Tria-GFF-Nap**) and validated that assembly could drive metal-free azide–alkyne cycloaddition reaction with excellent regioselectivity. Hydrogelation, TEM, and CD results showed that the nanoribbons in **Nap-FFK-Azi** + **Nap-FFG-Alk** hydrogel were similar to those in **Nap-FFK-Tria-GFF-Nap** solution. HPLC analyses indicated that, it was self-assembly but not other factors that drived the azide–alkyne cycloaddition between **Nap-FFK-Azi** and **Nap-FFG-Alk** to yield the pure product **Nap-FFK-Tria-GFF-Nap**. Real-time TEM observations validated that, **Nap-FFK-Azi** nanofibers were firstly formed in **Nap-FFK-Azi** + **Nap-FFG-Alk** hydrogel and then **Nap-FFG-Alk** is driven to react with the assemblies to yield the regionally selective product **Nap-FFK-Tria-GFF-Nap**. Previous studies have revealed that hydrophobic interactions and hydrogen-bonding could accelerate the Diers-Alder reaction^20^. This work demonstrated another example of using assembly to realize metal-free azide–alkyne cycloaddition with excellent regioselectivity. Currently, metal-free bioorthogonal click reactions show promising potential in a variety of biological applications including biomolecule labeling^21^, prodrug activation^22^, and cancer theranostics^23^. Encouraged by this work, we are applying supramolecular self-assembly to realize more reactions without metal ion catalysis, and those works are on the way.

## Methods

### Representative procedure for assembly-driven azide–alkyne cycloaddition

Nap-FFK-Azi (2.5 mmol, 1.0 equiv.) and Nap-FFG-Alk (2.5 mmol, 1.0 equiv.) were mixed in 1 mL PBS (10 mM, pH 9), thereafter the mixture solution was heated at 100 °C for 1 h and aging at 4 °C. During the period of aging, assembly-driven azide–alkyne cycloaddition evolved over time and afforded regioselevtive product.

## Supplementary information

Supplementary Information

## Data Availability

The authors declare that all the data supporting the findings of this study are available within the article and supplementary information and from the corresponding authors upon request. Source data are provided with this paper.

## Source data

Source Data
