# Supplementary material for: Assembly drives regioselective azide-alkyne cycloaddition reaction
Source: Nat Commun. 2023 Jul 4;14:3935. doi: 10.1038/s41467-023-39658-0 (PMC10319796; doi:10.1038/s41467-023-39658-0)
Supplement: Supplementary file 1 — Supplementary Information [file 41467_2023_39658_MOESM1_ESM.pdf]

# SUPPLEMENTARY INFORMATION

# Assembly drives regioselective azide-alkyne cycloaddition reaction

Qiaochu Jiang[†1], Wenjun Zhan[†1], Xiaoyang Liu[1], Lin Bai[1], Manli Wang[1], Ying Xu[1], and Gaolin Liang[★1]

[1]State Key Laboratory of Digital Medical Engineering, School of Biological Science and Medical Engineering, Southeast University, 2 Sipailou, Nanjing 210096, China

[†]These authors contributed equally to this work

[★]Correspondence and requests for materials should be addressed to e-mails: gliang@seu.edu.cn (G. L.).

## SUPPLEMENTRAY METHODS

### Materials and Instruments

All the starting materials were obtained from GL Biochem Co., Ltd. (Shanghai, China), Macklin Biochemical Co., Ltd (Shanghai, China), and Aladdin Chemistry Co., Ltd. (Shanghai, China). Commercially available reagents were used without further purification unless noted otherwise. All chemicals were reagent grade or better. Ultrapure water (18.2 MΩ·cm) was used throughout the experiment.

Ultraviolet-Visible (UV-Vis) absorption spectra were recorded on the Lambda 365 spectrometer (PerkinElmer, Germany). Transmission electron micrograph (TEM) images were obtained on a JEM-2100 transmission electron microscope (JEOL, Japan). High-performance liquid chromatography (HPLC) analyses were performed on an Agilent 1260 Infinity II Prime system equipped with a G1322A pump and an in-line diode array UV detector using an Agilent Zorbax 300SB-C18 RP column, with acetonitrile (0.1% of TFA) and water (0.1% of TFA) as the eluent (Agilent, USA). HPLC purification was performed on a 1290 Infinity system equipped with two LC-20AP pumps and an SPD-20A UV-Vis detector using a Shimazu PRC-ODS column (Agilent, USA). $^{1}$H-NMR and $^{13}$C-NMR spectra were measured on Avance III HD 600 MHz spectrometer (Bruker, Germany). Mass spectra (MS) were measured with Infinity Lab LC/MSD (Agilent, U.S.A.) and Ultraflextreme (Bruker Corporation, American). Circular dichroism (CD) spectra were obtained on a Chriascan qCD (Applied Photophysics, England). Rheology test was conducted by Haake RheoStress 6000 (Thermo Scientific), with cone-and plate geometry (1°/20 mm) at the gap of 370 μm.

### Preparation of molecular assemblies

A stock solution of **Nap-FFK-Azi**, **Nap-FFG-Alk**, **Nap-FF-Nva**, and **Nap-FFK-Tria-GFF-Nap** was prepared in PBS (pH = 9, 2.5 mM) and the self-assembly process was initiated by heating-cooling (100 °C - 4 °C) operation. **Nap-FFK-Azi** + **Nap-FFG-Alk** hydrogel was obtained from the solution equally mixed of **Nap-FFK-Azi** (pH = 9, 5 mM) and **Nap-FFG-Alk** (pH = 9, 5 mM) by heating-cooling operation**.** **Nap-FFK-Azi** + **Nap-FF-Nva** hydrogel was obtained from the solution equally mixed of **Nap-FFK-Azi** (pH = 9, 5 mM) and **Nap-FF-Nva** (pH = 9, 5 mM) by heating-cooling operation**.** The solutions of peptide were heated at 100 °C for 1 h, followed by aging at 4 °C for 24 h. Hydrogels were then achieved after several hours and can be stable for a long time under 4 °C.

### Critical concentration aggregation (CAC)

All the peptide solutions at concentrations of 2.5 mM to 5 μM were prepared in PBS (pH 9) for transmittance measuring by ultraviolet spectrophotometer, followed by calculating the intersection of dissolution.

### Transmission electron microscopy (TEM)

Samples were prepared by placing a drop of peptide dispersion (from hydrogels in 2.5 mM) on a carbon-coated Cu grid followed by the removal of excess solvent with natural withering.

### Circular dichroism (CD)

Data was recorded over a wavelength range of 190-260 nm with a bandwidth of 0.1 nm without thermocouple temperature control based on the self-assembly of 2.5 mM peptide

hydrogels dispersed in $Na_2CO_3$-$NaH_2PO_4$ buffer (pH = 9). Each dispersion was diluted ten-fold before collecting CD data.

**Synthesis and characterizations**

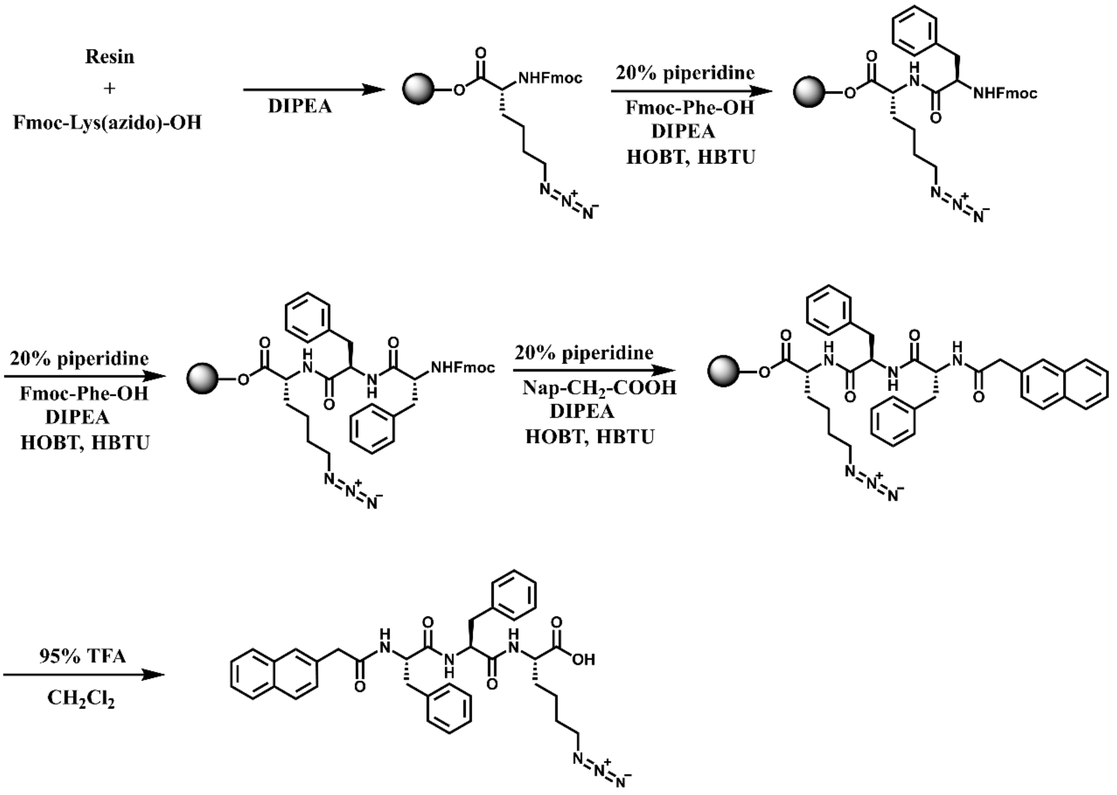


**Supplementary Figure 1**. Synthetic route of **Nap-FFK-Azi**

Synthesis of **Nap-FFK-Azi**: Compound Nap-Phe-Phe-Lys(azido)-OH (**Nap-FFK-Azi**) was synthesized by solid phase peptide synthesis (SPPS) and purified by HPLC. $^1$H NMR (300 MHz, $d_6$-DMSO) δ (ppm): 8.28 - 8.11 (m, 2 H), 7.94 - 7.67 (m, 3 H), 7.48 (m, 3 H), 7.31 - 7.12 (m, 10 H), 4.65 - 4.42 (m, 2 H), 4.28 - 4.14 (m, 1 H), 3.53 (dd, $J$ = 28.5, 14.1 Hz, 2 H), 3.32 (d, $J$ = 6.8 Hz, 3 H), 3.09 - 2.67 (m, 4 H), 1.79 - 1.31 (m, 6 H) (Supplementary Figure 3). $^{13}$C NMR (76 MHz, $d_6$-DMSO) δ (ppm): 171.65, 170.57, 138.40, 133.82, 129.95, 128.36, 127.01, 54.37, 52.46, 51.32, 43.01, 38.25, 28.36, 23.27 (Supplementary Figure 4). MS: calculated for **Nap-FFK-Azi** $[(M+H)^+]$: 635.29; obsvd. ESI-MS $[(M+H^+)]$: $m/z$ 635.3 (Supplementary Figure 5).

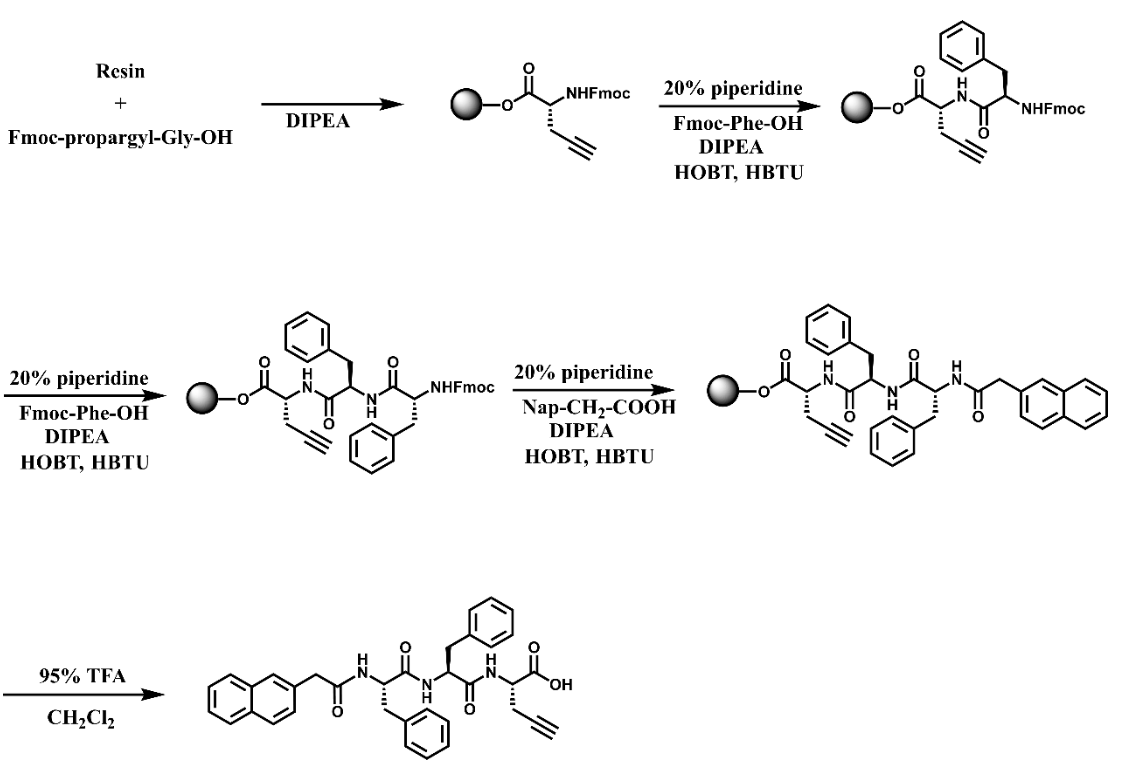


**Supplementary Figure 2**. Synthetic route of **Nap-FFG-Alk**

Synthesis of **Nap-FFG-Alk**: Compound Nap-Phe-Phe-Gly(alkynyl)-OH (**Nap-FFG-Alk**) was synthesized by SPPS and purified by HPLC. $^{1}$H NMR (500 MHz, $d_6$-DMSO) δ (ppm): 8.50 - 8.11 (m, 3 H), 7.92 - 7.68 (m, 3 H), 7.50 (dd, $J$ = 39.2, 32.2 Hz, 4 H), 7.26 - 7.13 (m, 10 H), 4.50 (m, 3 H), 3.54 (dd, $J$ = 41.6, 14.0 Hz, 2 H), 3.04 (dd, $J$ = 39.8, 12.9 Hz, 2 H), 2.90 (s, 1 H), 2.86 - 2.71 (m, 2 H), 2.64 (s, 2 H) (Supplementary Figure 6). $^{13}$C NMR (126 MHz, $d_6$-DMSO) δ (ppm): 172.00, 171.57, 170.25, 137.99, 133.49, 129.77, 128.52, 126.45, 80.71, 73.78, 54.22, 51.59, 42.72, 38.02, 21.68 (Supplementary Figure 7). MS: calculated for **Nap-FFG-Alk** [(M+H$^+$)]: 576.24; obsvd. ESI-MS [(M+H$^+$)]: $m/z$ 576.2 (Supplementary Figure 8).

Synthesis of **Nap-FFK-Tria-GFF-Nap**: Compound Nap-Phe-Phe-Lys(triazole)-Gly-Phe-Phe-Nap (**Nap-FFK-Tria-GFF-Nap)** was synthesized from: **Nap-FFG-Alk** (1 eq.) dissolved in DMF and mixture of $CuSO_4 \cdot 5H_2O$ (1 eq.) and sodium ascorbate (6 eq.) dissolved in ultrapure water were then added in the DMF solution contains **Nap-FFK-Azi**-Resin (1.5 eq.). The resulting mixture was kept at 25 °C for 6 h. The pure product was obtained after HPLC purification.$^{1}$H NMR (500 MHz, $d_6$-DMSO) δ (ppm): 8.46 - 8.15 (m, 6 H), 7.93 - 7.45 (m, 15 H), 7.25 - 7.13 (m, 20 H), 4.59 (d, $J$ = 6.4 Hz, 4 H), 4.25 (s, 4 H), 3.62 - 3.49 (m, 4 H), 3.12 - 2.66 (m, 10 H), 1.86 - 1.58 (m, 4 H), 1.30 (m, 2 H) (Supplementary Figure 9). $^{13}$C NMR (126 MHz, $d_6$-DMSO) δ (ppm): 171.44, 143.07, 137.82, 133.38, 129.94, 128.48, 126.41, 123.20, 54.01, 52.37, 49.60, 42.60, 37.94, 30.76, 29.85, 28.02, 22.96 (Supplementary Figure 10). MS: calculated for **Nap-FFK-Tria-GFF-Nap** [(M+H$^+$)]: 1210.53; obsvd. ESI-MS [(M+H$^+$)]: $m/z$ 1210.5 (Supplementary Figure 11).

Synthesis of **K-Tria-G**: Compound Fmoc-Lys(triazole)-Gly-Fmoc (**K-Tria-G)** was synthesized from: Fmoc-Lys(azido) (1 eq.) and Fmoc-Gly(alkynyl) (1.5 eq.) were dissolved in DMF, mixture of $CuSO_4 \cdot 5H_2O$ (1 eq.) and sodium ascorbate (6 eq.) dissolved in ultrapure water was then added. The mixture was kept at 25 °C for 6 h. The pure product was obtained after

HPLC purification. obsvd. ESI-MS [(M+H$^+$)]: *m/z* 730.3 (Supplementary Figure 28). $^{1}$H NMR (500 MHz, $d_6$-DMSO) δ (ppm): 7.92 - 7.68 (m, 10 H), 7.63 (d, *J* = 8.0 Hz, 1 H), 7.42 (s, 4 H), 7.34 (d, *J* = 7.1 Hz, 4 H), 4.25 (dd, *J* = 18.7, 6.7 Hz, 9 H), 3.96 (s, 1 H), 3.36 - 2.70 (m, 2 H), 1.86 - 1.48 (m, 4 H), 1.33 (m, 2 H) (Supplementary Figure 29). $^{13}$C NMR (126 MHz, $d_6$-DMSO) δ (ppm): 174.30, 173.42, 156.67, 156.44, 144.33, 144.29, 144.26, 141.21, 128.11, 127.55, 125.73, 120.57, 66.19, 66.10, 54.36, 54.16, 51.02, 49.56, 47.18, 47.09, 40.58, 40.49, 40.42, 40.33, 40.25, 40.16, 40.08, 39.99, 39.82, 39.66, 39.49, 30.65, 29.91, 28.32, 27.80, 23.40, 23.15 (Supplementary Figure 30).

Synthesis of **Nap-FFG-Nva**: Compound Nap-Phe-Phe-Nva-OH (**Nap-FF-Nva**) was synthesized by SPPS and purified by HPLC. $^{1}$H NMR (500 MHz, $d_6$-DMSO) δ (ppm): 8.29 - 8.12 (m, 3 H), 7.92 - 7.39 (m, 7 H), 7.20 (m, 10 H), 4.67 - 4.52 (m, 2 H), 4.26 (s, 1 H), 3.61 - 3.48 (m, 2 H), 3.25 - 2.91 (dd, *J* = 42.5, 13.9 Hz, 2 H), 2.88 - 2.71 (m, 2 H), 1.78 - 1.55 (m, 2 H), 1.40 - 1.29 (m, 2 H), 0.90 - 0.86 (m, 3 H) (Supplementary Figure 31). $^{13}$C NMR (126 MHz, $d_6$-DMSO) δ (ppm): 174.00, 171.51, 170.27, 138.24, 134.36, 133.42, 132.22, 129.76, 129.71, 128.50, 128.38, 128.08, 127.92, 127.85, 127.73, 126.73, 126.62, 126.43, 125.92, 54.26, 54.06, 52.11, 42.72, 39.84, 39.68, 33.65, 19.07, 14.01 (Supplementary Figure 32). MS: calculated for **Nap-FFG-Nva** [(M-H)$^-$]: 578.26; obsvd. ESI-MS [(M-H)$^-$]: *m/z* 578.3 (Supplementary Figure 33).

Synthesis of **Nap-FFK$_d$-Azi**: Compound Nap-Phe-Phe-(D-Lys(azido))-OH (**Nap-FFK$_d$-Azi**) was synthesized by SPPS and purified by HPLC. $^{1}$H NMR (500 MHz, $d_6$-DMSO) δ (ppm): 8.32 - 8.16 (m, 3 H), 7.88 - 7.41 (m, 7 H), 7.24 - 7.14 (m, 10 H), 4.76 - 4.52 (m, 2 H), 4.22 (m, 1 H), 3.57 (d, *J* = 25.1 Hz, 2 H), 3.25 (m, 2 H), 3.02 - 2.88 (m, 2 H), 2.81-2.48 (dd, *J* = 41.2, 23.7 Hz, 2 H), 1.68 - 1.42 (m, 4 H), 1.25 - 1.18 (m, 2 H) (Supplementary Figure 40). $^{13}$C NMR (126 MHz, $d_6$-DMSO) δ (ppm): $^{13}$C NMR (126 MHz, DMSO) δ 173.83, 171.36, 171.19, 170.36, 138.22, 137.88, 134.37, 133.43, 132.23, 129.73, 128.51, 128.41, 128.08, 127.94, 127.91, 127.84, 127.74, 126.76, 126.61, 126.43, 125.92, 54.32, 52.06, 50.95, 42.74, 39.65, 38.72, 31.18, 28.29, 22.91 (Supplementary Figure 41). MS: calculated for **Nap-FFK$_d$-Azi** [(M-H)$^-$]: 633.3; obsvd. ESI-MS [(M-H$^-$)]: *m/z* 633.28 (Supplementary Figure 42).

Synthesis of **Nap-FFG$_d$-Alk**: Compound Nap-Phe-Phe-(D-Gly(alkynyl))-OH (**Nap-FFG$_d$-Alk**) was synthesized by SPPS and purified by HPLC. $^{1}$H NMR (500 MHz, $d_6$-DMSO) δ (ppm): 8.49 - 8.47 (d, *J* = 7.9 Hz, 1 H), 8.21 - 8.17 (dd, *J* = 20.7, 8.4 Hz, 2 H), 7.92 - 7.41 (m, 7 H), 7.26 - 7.14 (m, 10 H), 4.77 - 4.39 (m, 3 H), 3.55 (dd, *J* = 41.0, 14.1 Hz, 2 H), 3.03 - 2.94 (dd, *J* = 17.2, 13.8, 4.0 Hz, 2 H), 2.91 - 2.81 (m, 2 H), 2.75 (dd, *J* = 13.6, 10.3 Hz, 1H), 2.66 - 2.54 (m, 2H) (Supplementary Figure 43). $^{13}$C NMR (126 MHz, $d_6$-DMSO) δ (ppm): 172.02, 171.47, 171.38, 170.25, 138.25, 137.95, 134.37, 133.42, 132.22, 129.79, 129.74, 128.50, 128.40, 128.08, 127.92, 127.85, 127.73, 126.76, 126.60, 126.44, 125.92, 80.62, 73.67, 54.23, 54.03, 51.36, 42.72, 38.47, 38.06, 21.85 (Supplementary Figure 44). MS: calculated for **Nap-FFG-Alk** [(M-H)$^-$]: 574.23; obsvd. ESI-MS [(M-H)$^-$]: *m/z* 574.2 (Supplementary Figure 45).

Synthesis of **Nap-F$_d$F$_d$K-Azi**: Compound Nap-(D-Phe)-(D-Phe)-Lys(azido)-OH (**Nap-F$_d$F$_d$K-Azi**) was synthesized by SPPS and purified by HPLC. $^{1}$H NMR (500 MHz, $d_6$-DMSO) δ (ppm): 8.31 - 8.16 (m, 3 H), 7.92 - 7.37 (m, 7 H), 7.23 - 7.14 (m, 10 H), 4.74 - 4.54 (m, 2 H), 4.20 (d, *J* = 8.4, 5.0 Hz, 1 H), 3.60 - 3.50 (m, 2 H), 3.26 (m, 2 H), 3.01 - 2.95 (m, 2 H), 2.89 - 2.73 (m, 2 H), 1.66 - 1.41 (m, 4 H), 1.22 (dd, *J* = 15.6, 7.6 Hz, 2 H) (Supplementary Figure 46). $^{13}$C NMR (126 MHz, $d_6$-DMSO) δ (ppm): 173.83, 171.34, 171.17, 170.32, 138.22, 137.88,

134.38, 133.42, 132.22, 129.73, 128.50, 128.40, 128.08, 127.93, 127.91, 127.84, 127.73, 126.75, 126.60, 126.43, 125.91, 54.30, 52.05, 50.95, 42.73, 39.51, 38.00, 31.18, 28.29, 22.90 (Supplementary Figure 47). MS: calculated for **Nap-F$_d$F$_d$K-Azi** [(M-H)$^-$]: 633.3; obsvd. ESI-MS [(M-H)$^-$]: *m/z* 633.28 (Supplementary Figure 48).

Synthesis of **Nap-F$_d$F$_d$G-Alk**: Compound Nap-(D-Phe)-(D-Phe)-Gly(alkynyl)-OH (**Nap-F$_d$F$_d$G-Alk**) was synthesized by SPPS and purified by HPLC. $^1$H NMR (500 MHz, $d_6$-DMSO) δ (ppm): 8.49 (d, $J$ = 7.8 Hz, 1 H), 8.21 (dd, $J$ = 20.5, 8.3 Hz, 2 H), 7.90 - 7.63 (m, 3 H), 7.63 - 7.39 (m, 4 H), 7.24 - 7.13 (m, 10 H), 4.73 - 4.38 (m, 3 H), 3.55 (d, $J$ = 26.8 Hz, 2 H), 3.18 (s, 1 H), 3.03 (dd, $J$ = 31.4, 13.6 Hz, 2 H), 2.90 - 2.80 (m, 2 H), 2.77 - 2.58 (m, 2 H) (Supplementary Figure 49). $^{13}$C NMR (126 MHz, $d_6$-DMSO) δ (ppm): $^{13}$C NMR (126 MHz, DMSO) δ 172.03, 171.46, 171.38, 170.24, 138.25, 137.95, 134.37, 133.42, 132.22, 129.79, 129.74, 128.50, 128.40, 128.08, 127.92, 127.85, 127.72, 126.76, 126.60, 126.43, 125.92, 80.62, 73.67, 54.23, 54.03, 51.37, 42.72, 38.48, 38.06, 21.86 (Supplementary Figure 50). MS: calculated for **Nap-F$_d$F$_d$G-Alk** [(M-H)$^-$]: 574.2; obsvd. ESI-MS [(M-H)$^-$]: *m/z* 574.23 (Supplementary Figure 51).

Synthesis of **Nap-F$_d$F$_d$K$_d$-Azi**: Compound Nap-(D-Phe)-(D-Phe)-(D-Lys(azido))-OH (**Nap-F$_d$F$_d$K$_d$-Azi**) was synthesized by SPPS and purified by HPLC. $^1$H NMR (500 MHz, $d_6$-DMSO) δ (ppm): $^1$H NMR (500 MHz, DMSO) δ 8.32 - 8.10 (m, 3 H), 7.64 (dd, $J$ = 28.5, 7.2 Hz, 7 H), 7.25 - 7.11 (m, 11 H), 4.74 - 4.46 (m, 2 H), 4.24 (dd, $J$ = 12.9, 7.7 Hz, 1 H), 3.61 - 3.49 (m, 2 H), 3.29 (d, $J$ = 6.8 Hz, 2 H), 2.91 (m, 4 H), 1.76 – 1.33 (m, 6H) (Supplementary Figure 52). $^{13}$C NMR (126 MHz, $d_6$-DMSO) δ (ppm): 184.52, 173.79, 171.53, 170.20, 138.27, 138.07, 134.35, 133.42, 132.22, 129.76, 129.69, 128.50, 128.39, 128.08, 127.92, 127.85, 127.73, 126.74, 126.62, 126.43, 125.92, 54.29, 52.16, 51.00, 43.59, 38.00, 31.09, 29.41, 29.06, 23.08 (Supplementary Figure 53). MS: calculated for **Nap-F$_d$F$_d$K$_d$-Azi** [(M+H)$^+$]: 635.3; obsvd. ESI-MS [(M+H)$^+$]: *m/z* 635.1 (Supplementary Figure 54).

Synthesis of **Nap-F$_d$F$_d$G$_d$-Alk**: Compound Nap-(D-Phe)-(D-Phe)-(D-Gly(alkynyl))-OH (**Nap-F$_d$F$_d$G$_d$-Alk**) was synthesized by SPPS and purified by HPLC. $^1$H NMR (500 MHz, $d_6$-DMSO) δ (ppm):8.43 (d, $J$ = 7.7 Hz, 1 H), 8.22 (dd, $J$ = 29.9, 8.4 Hz, 2 H), 7.89 - 7.73 (m, 3 H), 7.63 - 7.41 (m, 4 H), 7.26 - 7.14 (m, 10 H), 4.68 - 4.37 (m, 3 H), 3.54 (dd, $J$ = 41.6, 14.1 Hz, 2 H), 3.10 - 2.99 (m, 2 H), 2.91 (s, 1 H), 2.85 - 2.72 (m, 2 H), 2.64 (d, $J$ = 5.9 Hz, 2 H) (Supplementary Figure 55). $^{13}$C NMR (126 MHz, $d_6$-DMSO) δ (ppm): 172.00, 171.54, 171.43, 170.25, 138.27, 137.98, 134.36, 133.50, 132.22, 129.76, 129.72, 128.51, 128.40, 128.08, 127.92, 127.85, 127.73, 126.76, 126.62, 126.44, 125.92, 80.70, 73.77, 54.21, 54.10, 51.59, 42.72, 39.51, 21.68 (Supplementary Figure 56). MS: calculated for **Nap-F$_d$F$_d$G$_d$-Alk** [(M+H)$^+$]: 576.2; obsvd. ESI-MS [(M+H)$^+$]: *m/z* 576.1 (Supplementary Figure 57).

**Nap-FFK$_d$-Tria-G$_d$FF-Nap**, **Nap-F$_d$F$_d$K-Tria-GF$_d$F$_d$-Nap**, and **Nap-F$_d$F$_d$K$_d$-Tria-G$_d$F$_d$F$_d$-Nap** were obtained from the same synthesis route of **Nap-FFK-Tria-GFF-Nap**, and purified by HPLC and characterized by ESI-MS (Supplementary Figures 58-60).

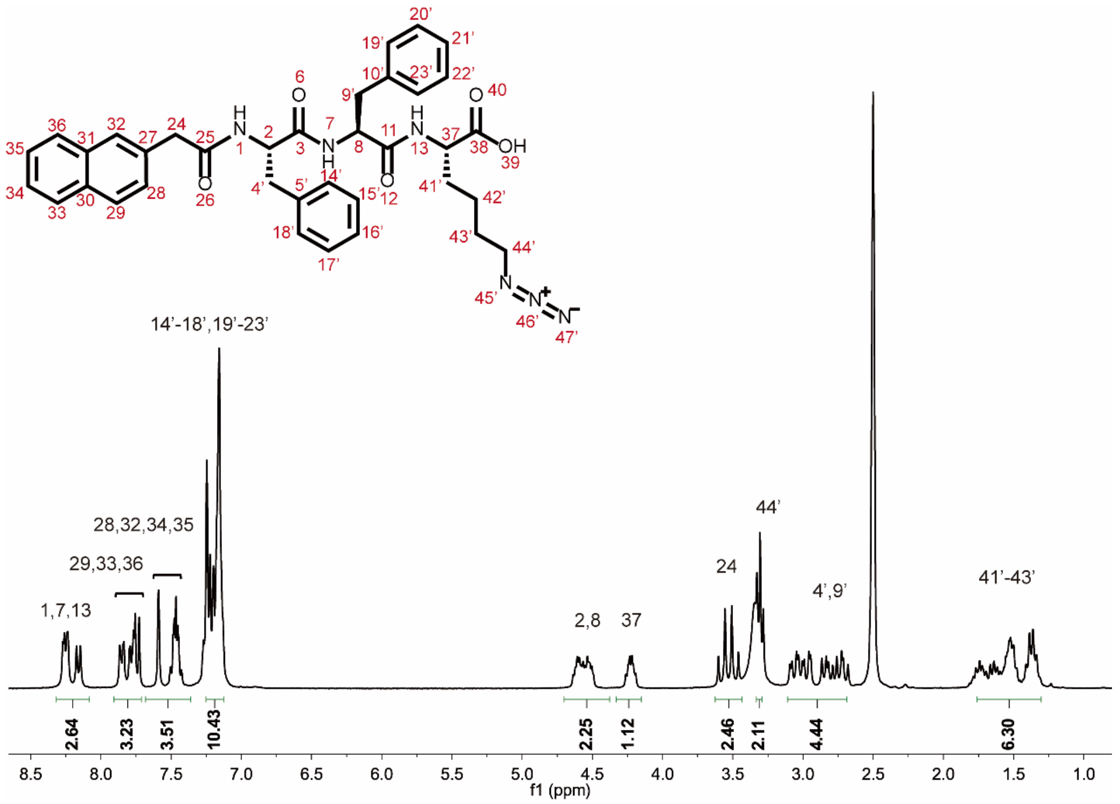


**Supplementary Figure 3.** $^{1}$H NMR (300 MHz, 25 °C) spectrum of **Nap-FFK-Azi** in $d_6$-DMSO.

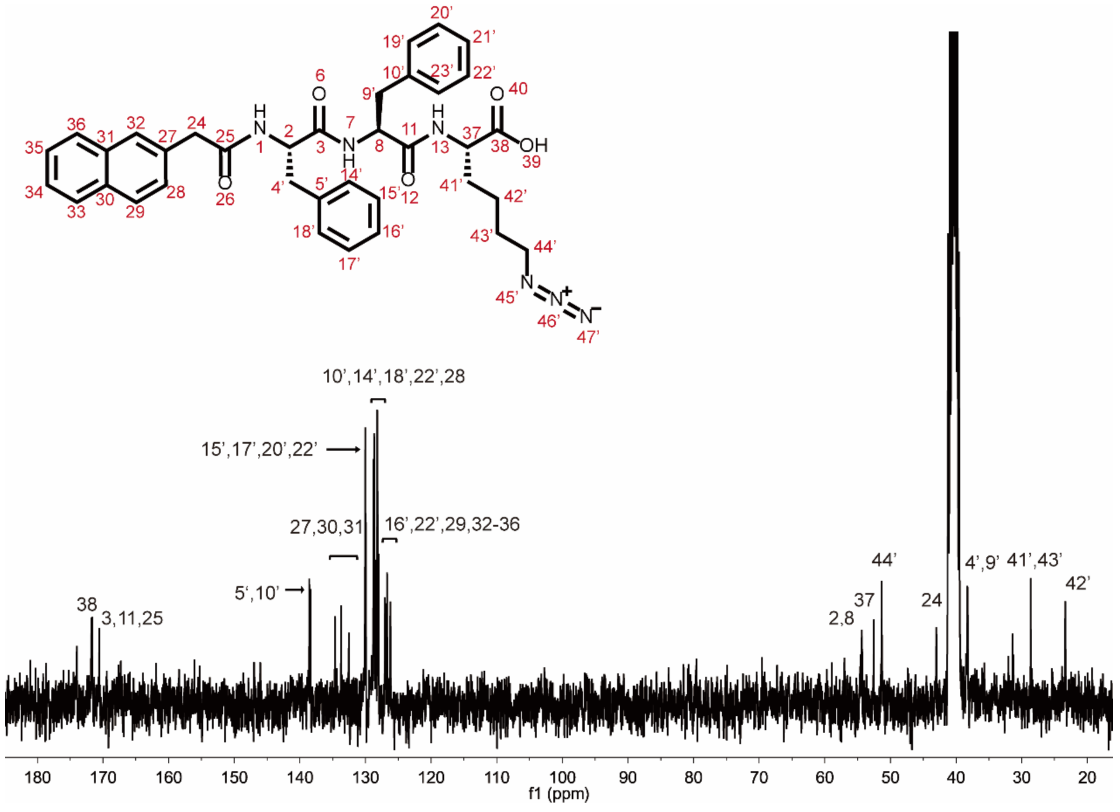


**Supplementary Figure 4.** $^{13}$C NMR (76 MHz, 25 °C) spectrum of **Nap-FFK-Azi** in $d_6$-DMSO.

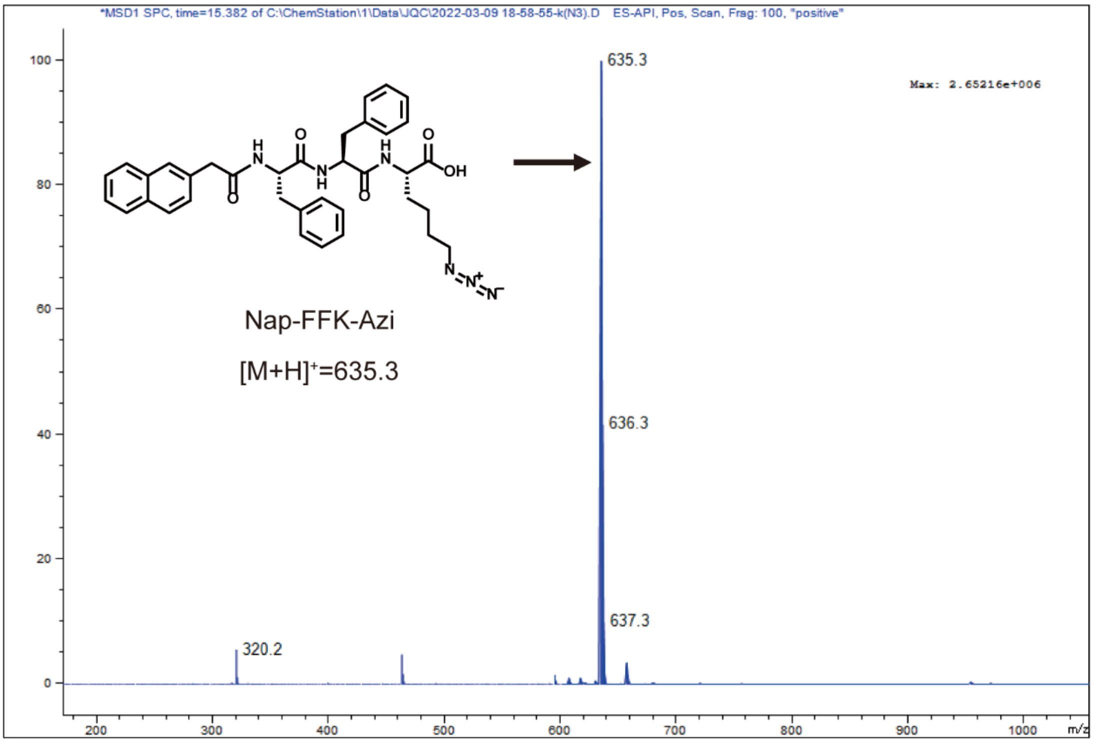


**Supplementary Figure 5.** ESI-MS spectrum of **Nap-FFK-Azi**.

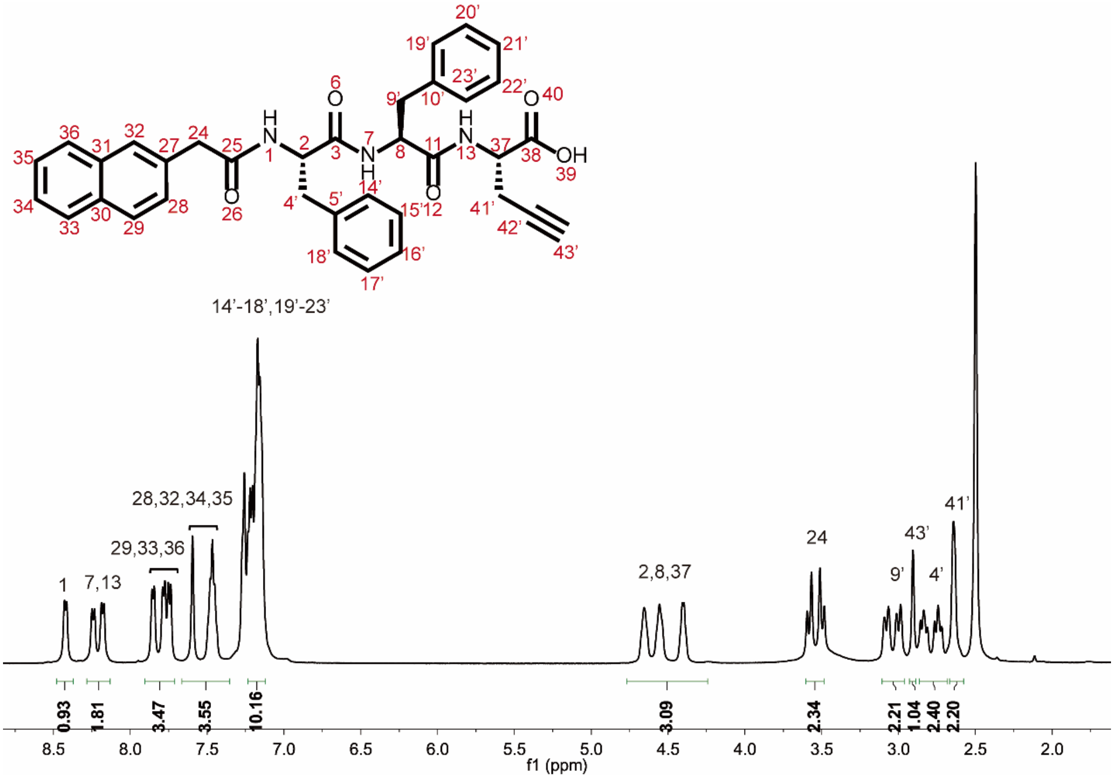


**Supplementary Figure 6.** $^{1}$H NMR (500 MHz, 25 °C) spectrum of **Nap-FFG-Alk** in $d_6$-DMSO.

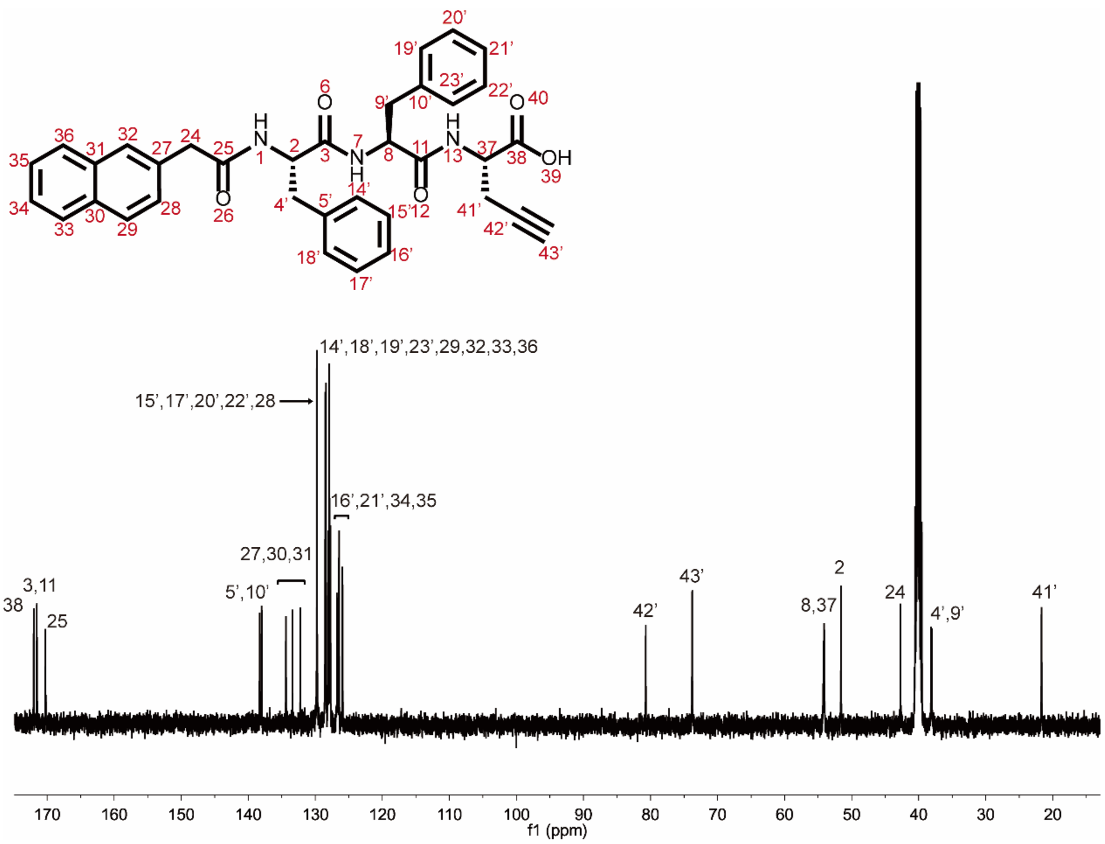


**Supplementary Figure 7.** $^{13}$C NMR (126 MHz, 25 °C) spectrum of **Nap-FFG-Alk** in $d_6$-DMSO.

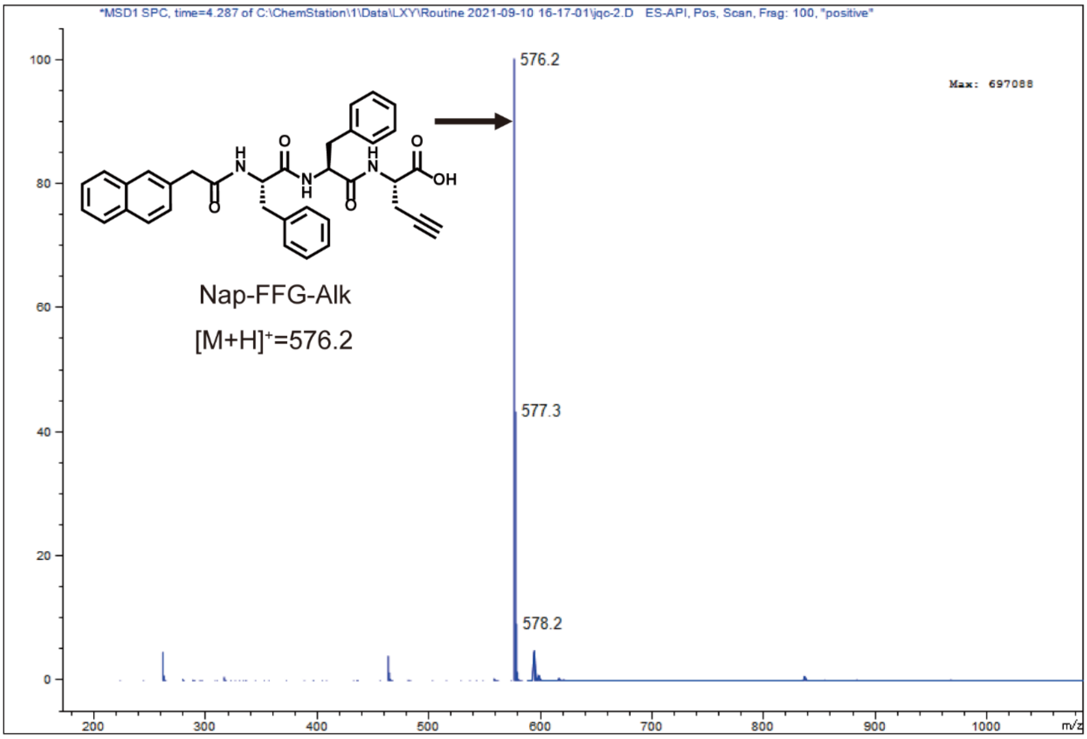


**Supplementary Figure 8.** ESI-MS spectrum of **Nap-FFG-Alk**.

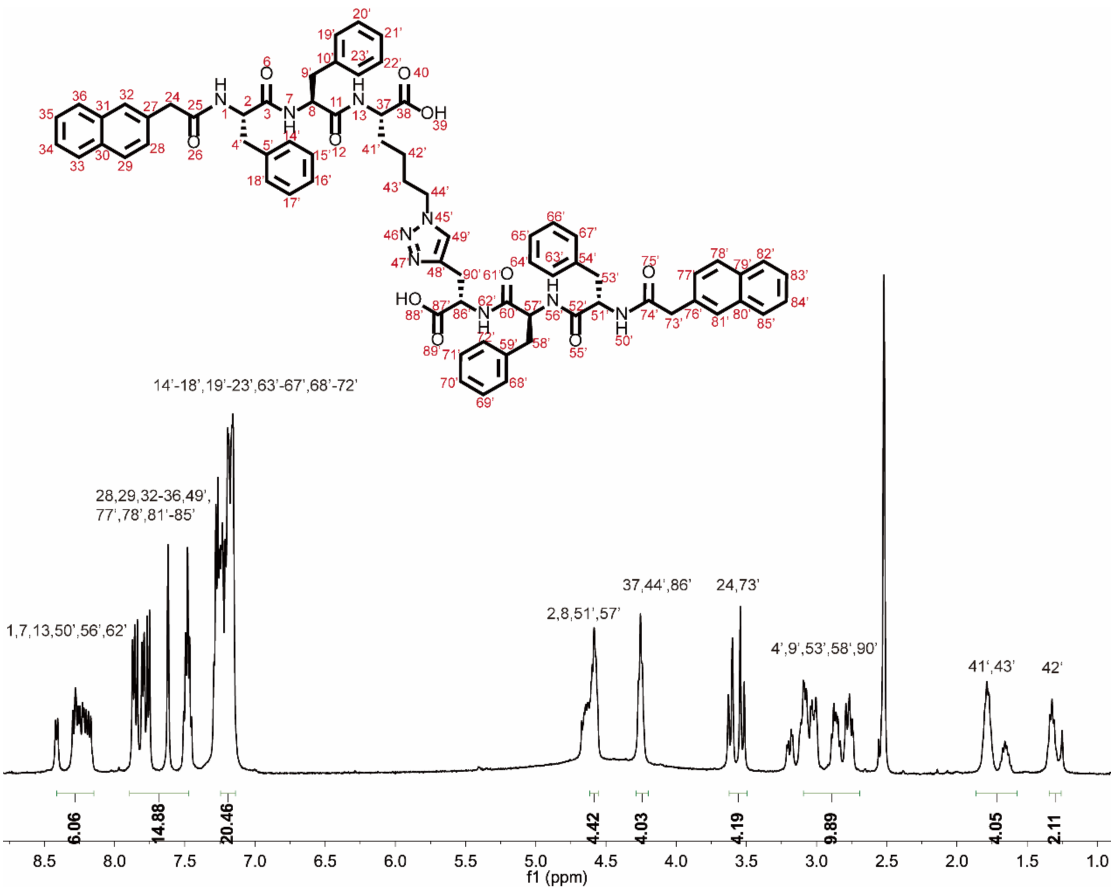


**Supplementary Figure 9.** $^{1}$H NMR (500 MHz, 25 °C) spectrum of **Nap-FFK-Tria-GFF-Nap** in $d_6$-DMSO.

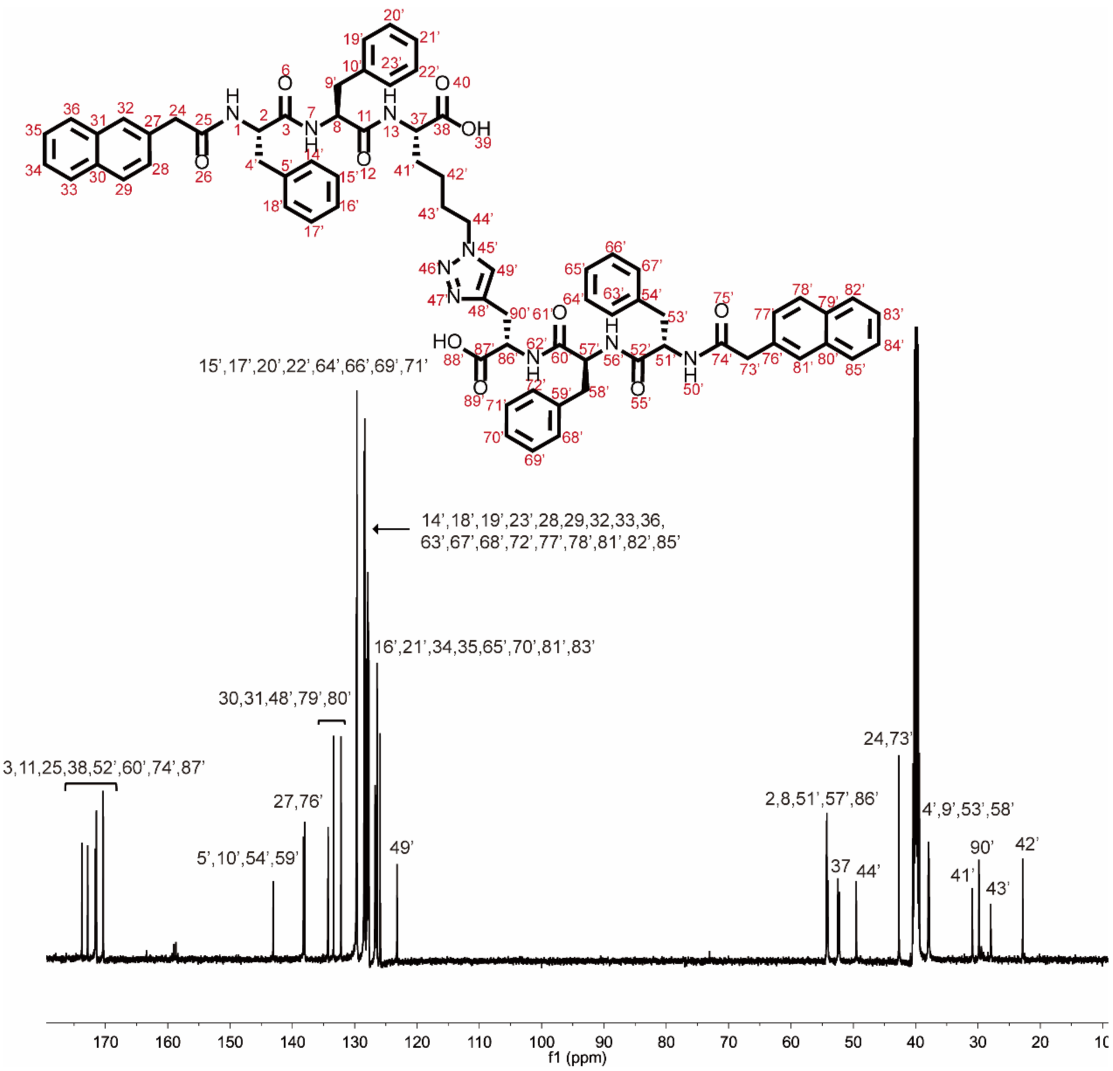


**Supplementary Figure 10.** $^{13}C$ NMR (126MHz, 25 °C) spectrum of **Nap-FFK-Tria-GFF-Nap** in $d_6$-DMSO.

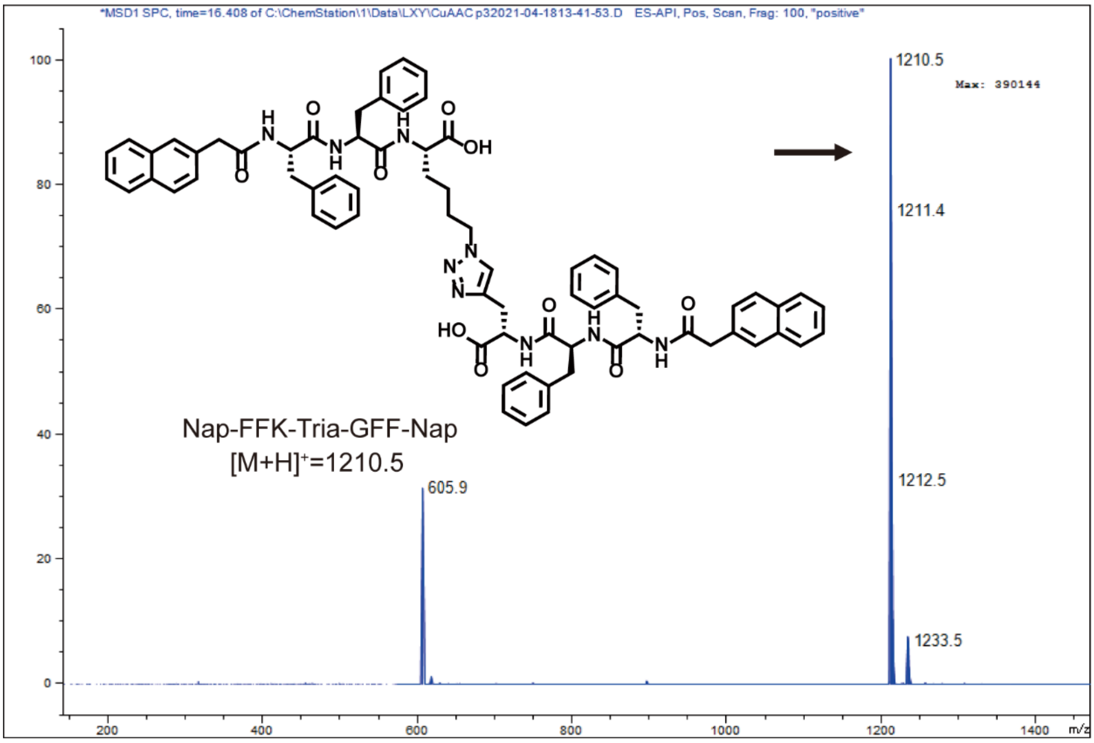


**Supplementary Figure 11.** ESI-MS spectrum of **Nap-FFK-Tria-GFF-Nap**.

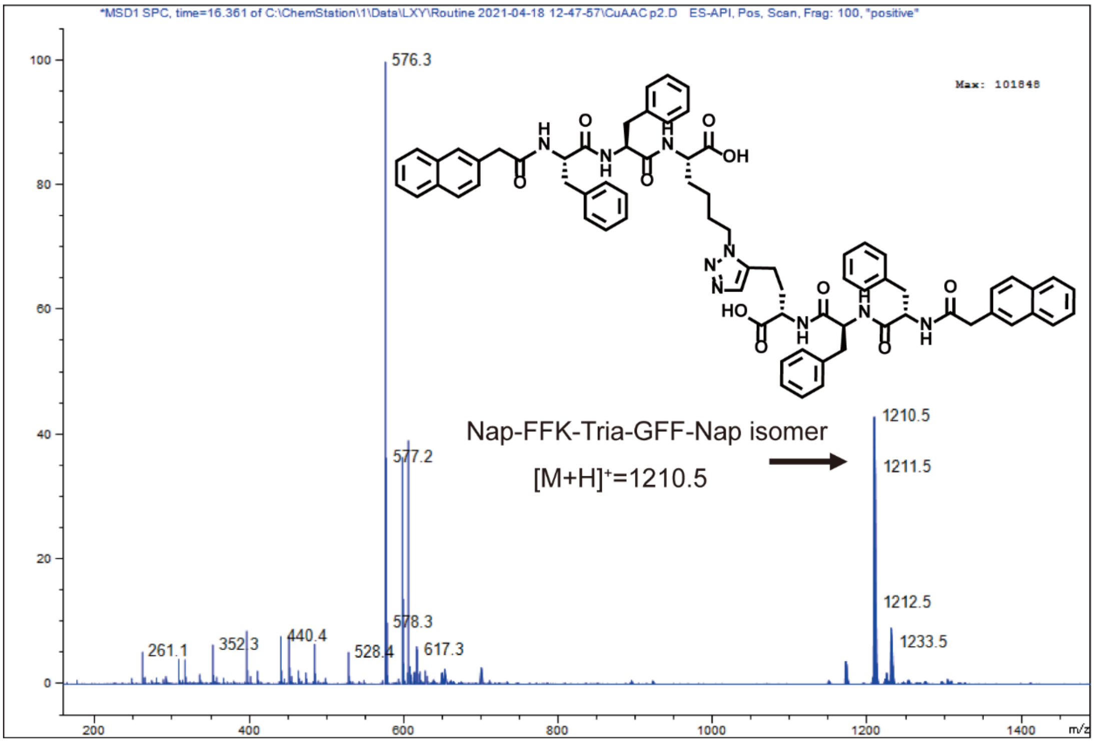


**Supplementary Figure 12.** ESI-MS spectrum of the 1st peak in Figure 1b.

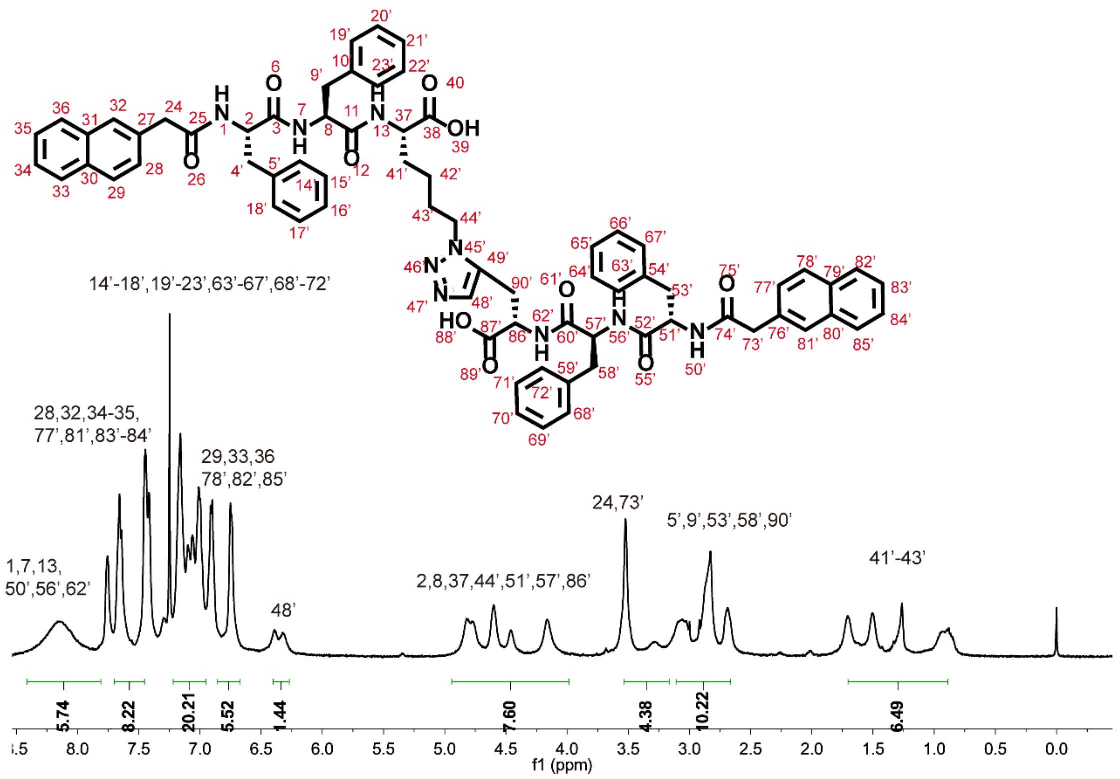


**Supplementary Figure 13.** $^{1}H$ NMR (500 MHz, 25 °C) spectrum of **Nap-FFK-Tria-GFF-Nap** isomer in $CDCl_3$.

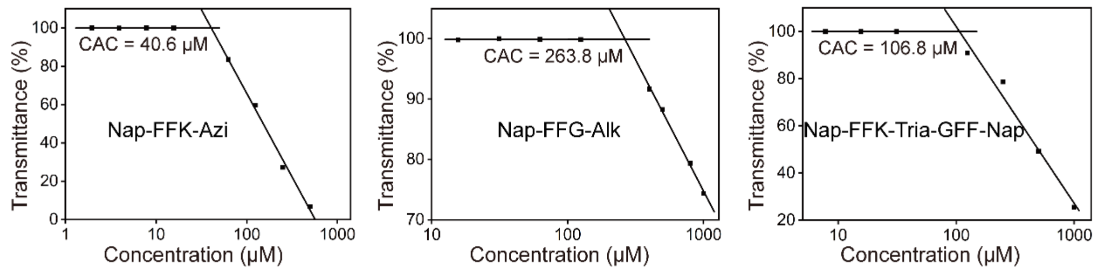


**Supplementary Figure 14.** Critical aggregation concentrations (CACs) of **Nap-FFK-Azi**, **Nap-FFG-Alk**, and **Nap-FFK-Tria-GFF-Nap**.

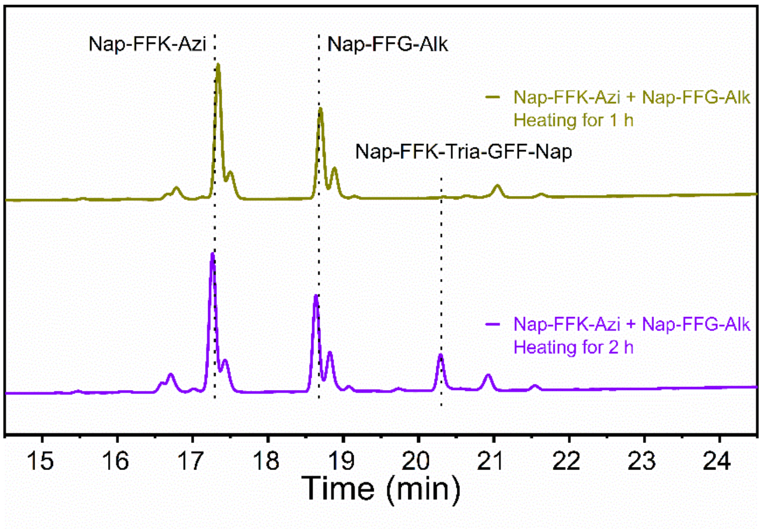


**Supplementary Figure 15.** HPLC traces of 2.5 mM **Nap-FFK-Azi** + **Nap-FFG-Alk** heating for 1 h (grayish) and 2.5 mM **Nap-FFK-Azi** + **Nap-FFG-Alk** heating for 2 h (violet). Wavelength for detection: 254 nm.

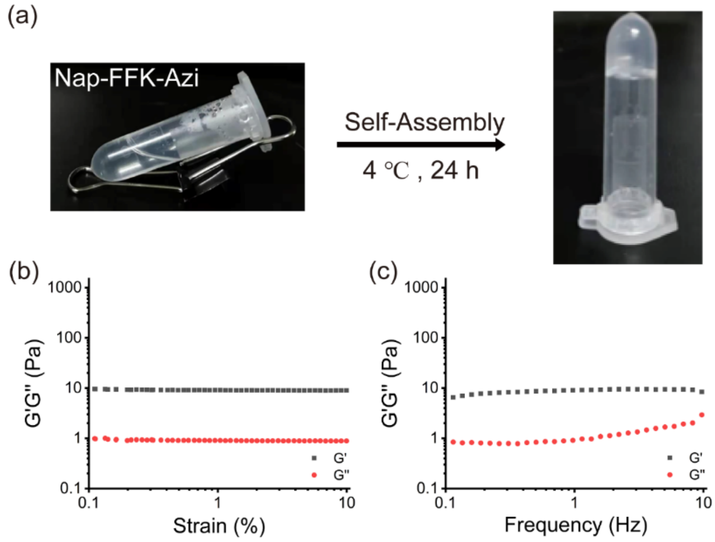


**Supplementary Figure 16.** Photographs of (a) the solution of **Nap-FFK-Azi** and its self-assembled hydrogel at 0.15 wt% (2.5 mM) and pH = 9 over 24 h. (b) Strain dependence of the dynamic storage moduli (G′) and the loss moduli (G″) of **Nap-FFK-Azi** hydrogel (4 °C, frequency: 1 Hz). (c) Frequency dependence of the dynamic storage moduli (G′) and the loss moduli (G″) of **Nap-FFK-Azi** hydrogel. (4 °C, strain: 1.0%).

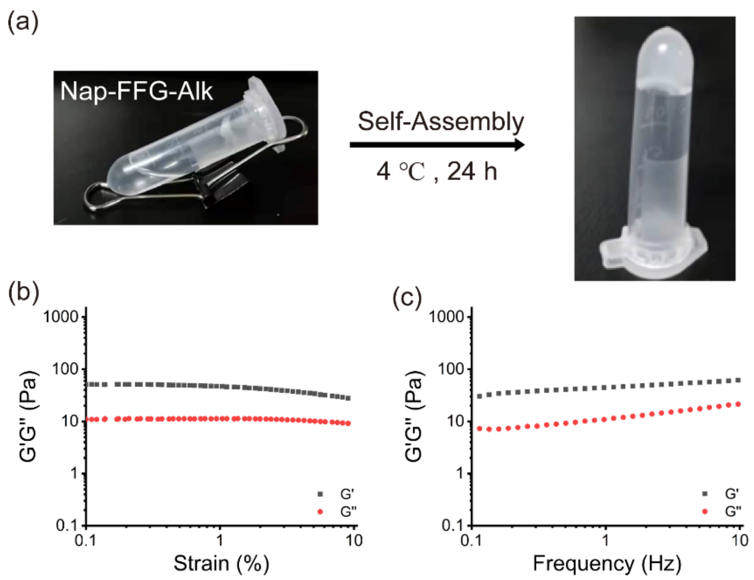


**Supplementary Figure 17.** Photographs of (a) the solution of **Nap-FFG-Alk** and its self-assembled hydrogel at 0.32 wt% (2.5 mM) and pH = 9 over 24 h. (b) Strain dependence of the dynamic storage moduli (G′) and the loss moduli (G″) of **Nap-FFG-Alk** hydrogel (4 °C, frequency: 1 Hz). (c) Frequency dependence of the dynamic storage moduli (G′) and the loss moduli (G″) of Nap-FFG-Alk hydrogel. (4 °C, strain: 1.0%).

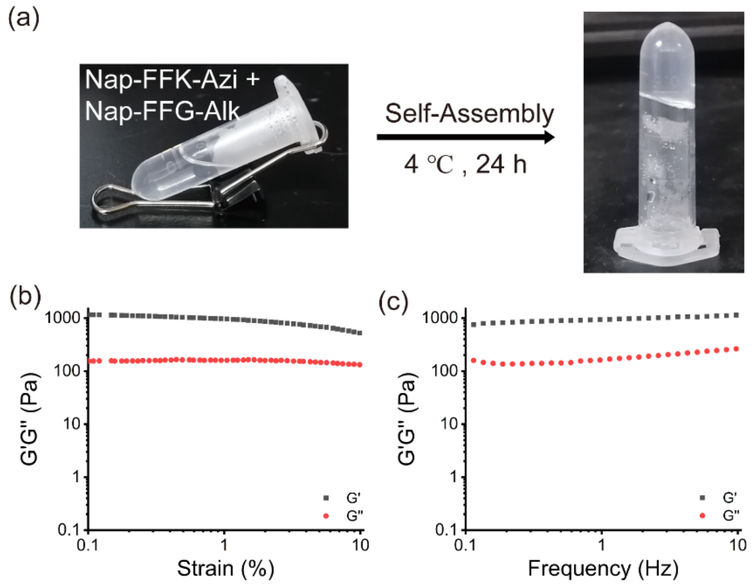


**Supplementary Figure 18.** Photographs of (a) the solution of co-assembly mixture (equal volume **Nap-FFK-Azi** (2.5 mM / 0.15 wt%) and **Nap-FFG-Alk** (2.5 mM / 0.32 wt%) and its self-assembled hydrogel at pH = 9 over 24 h. (b) Strain dependence of the dynamic storage moduli (G′) and the loss moduli (G″) of co-assembly mixture (4 °C, frequency: 1 Hz). (c) Frequency dependence of the dynamic storage moduli (G′) and the loss moduli (G″) of co-assembly mixture hydrogel. (4 °C, strain: 1.0%).

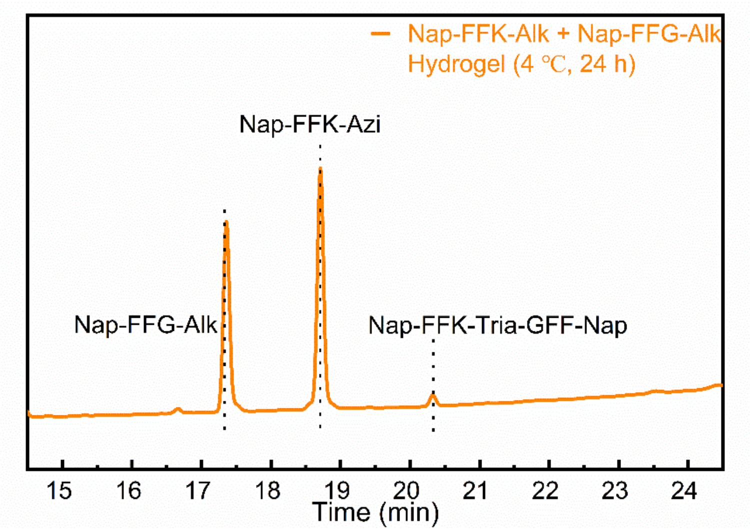


**Supplementary Figure 19.** HPLC trace of 2.5 mM **Nap-FFK-Azi** + **Nap-FFG-Alk** hydrogel (orange) in PBS (PH 9). The hydrogel was obtained after the solution was heated at 100 °C for 1 h and then cooled at 4 °C for 24 h. Wavelength for detection: 254 nm.

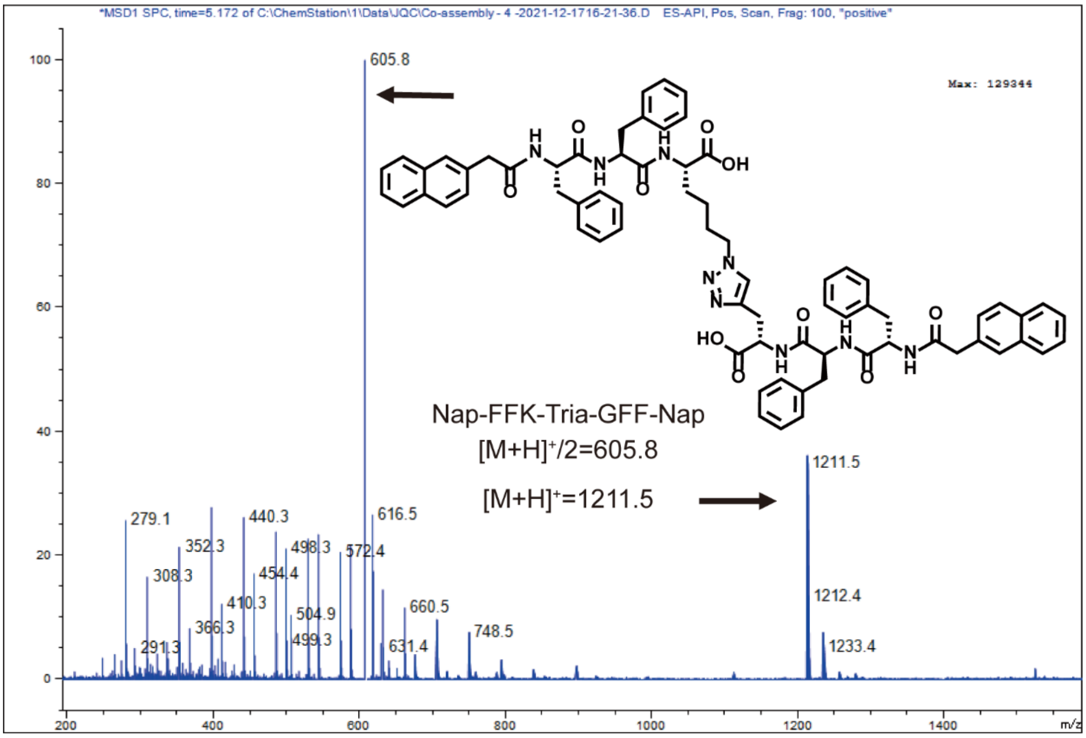


**Supplementary Figure 20.** ESI-MS spectrum of the cycloaddition reaction product (retention time 20.3 min) in Figure 2a.

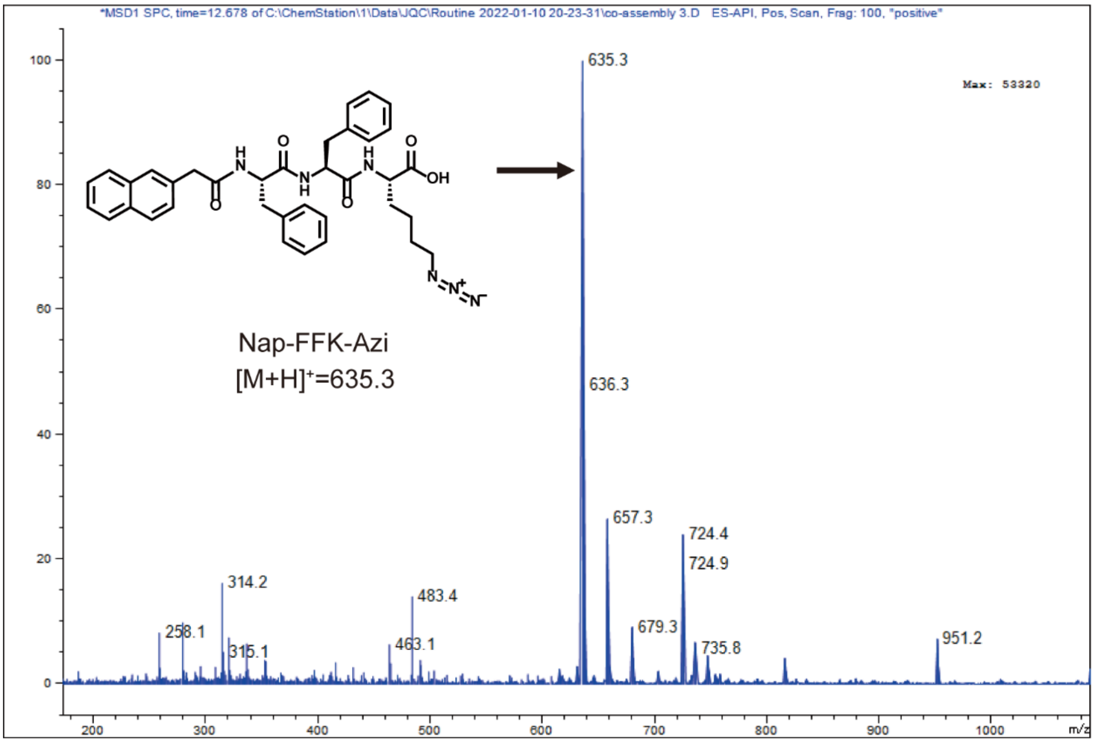


**Supplementary Figure 21.** ESI-MS spectrum of the peak at 17.5 min in Figure 3a.

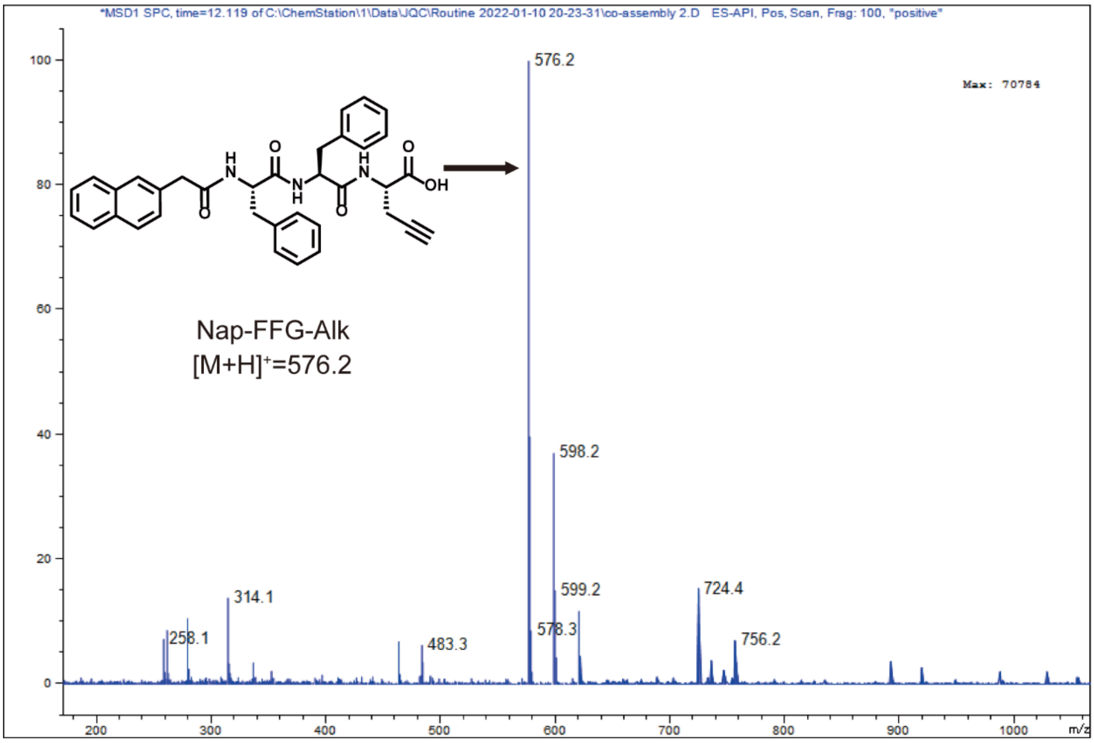


**Supplementary Figure 22.** ESI-MS spectrum of the peak at 18.7 min in Figure 3a.

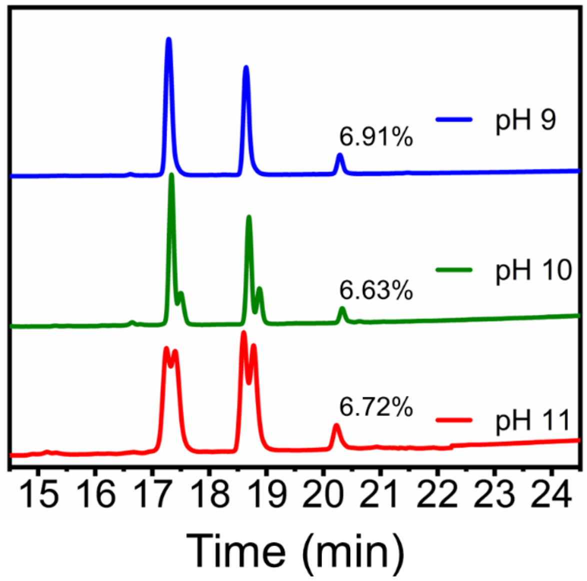


**Supplementary Figure 23.** HPLC traces of 2.5 mM **Nap-FFK-Azi** + **Nap-FFG-Alk** hydrogel in PBS at pH 9 (blue), pH 10 (green), or pH 11 (red), respectively. All the hydrogels were obtained after the solutions were heated at 100 ℃ for 1 h and then cooled at 4 ℃ for 5 d. Wavelength for detection: 254 nm.

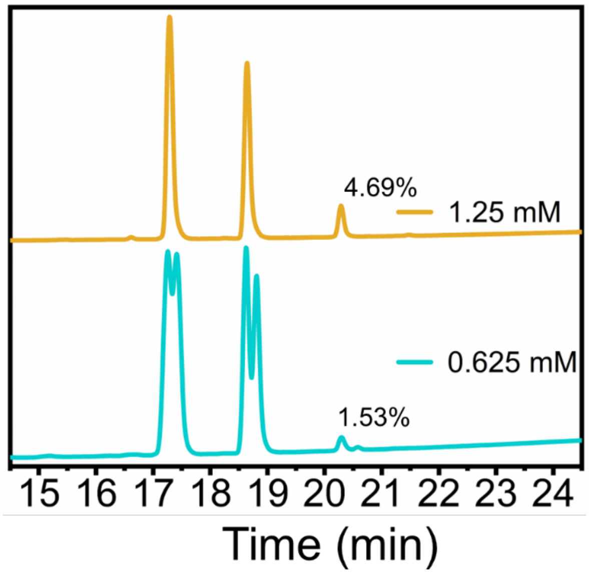


**Supplementary Figure 24.** HPLC traces of 1.25 mM **Nap-FFK-Azi** + **Nap-FFG-Alk** hydrogel (yellow) and 0.625 mM **Nap-FFK-Azi** + **Nap-FFG-Alk** hydrogel (aquamarine blue) in PBS at pH 9. All the hydrogels were obtained after the solutions were heated at 100 ℃ for 1 h and then cooled at 4 ℃ for 5 d. Wavelength for detection: 254 nm.

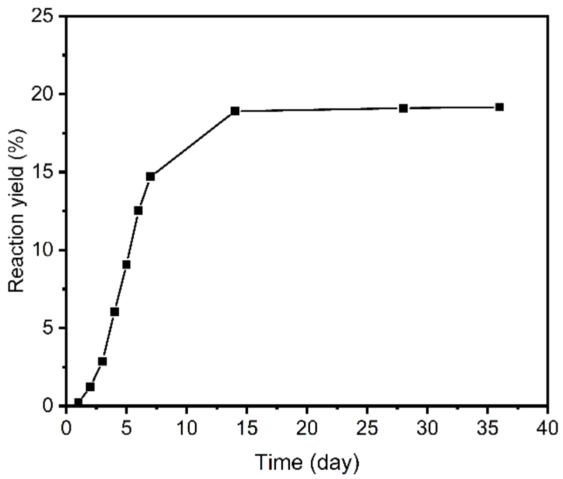


**Supplementary Figure 25.** Time course reaction yield of the assembly-driven azide-alkyne cycloaddition reaction in **Nap-FFK-Azi** + **Nap-FFG-Alk** hydrogel in PBS at pH 9.

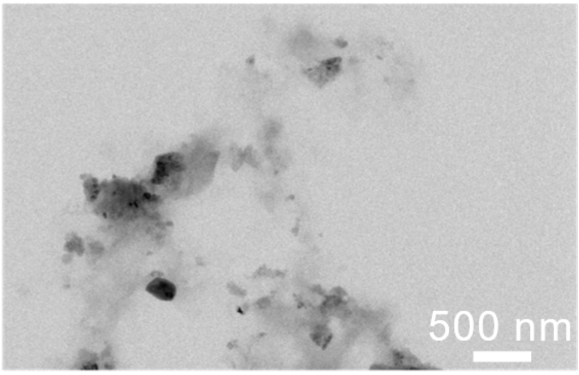


**Supplementary Figure 26.** TEM image of 2.5 mM **K-Azi** + **G-Alk** in PBS at 7 d. scale bar = 500 nm.

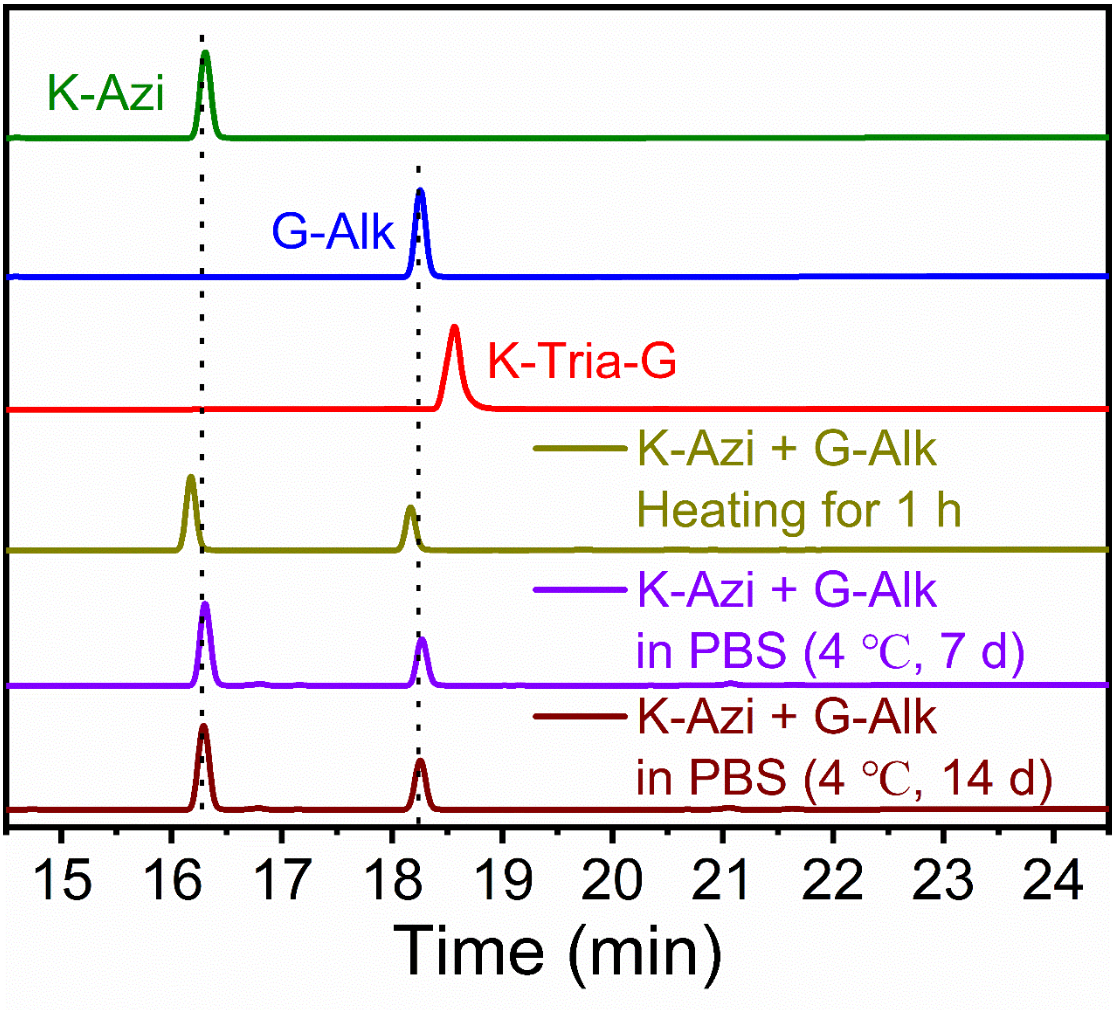


**Supplementary Figure 27.** HPLC traces of 2.5 mM **K-Azi** (green), 2.5 mM **G-Alk** (blue), 2.5 mM **K-Tria-G** (red), 2.5 mM **K-Azi** + **G-Alk** heating for 1 h (dark yellow), 2.5 mM **K-Azi** + **G-Alk** 7 d post in 4 °C after 1 h heating (purple) and 2.5 mM **K-Azi** + **G-Alk** 14 d post in 4 °C after 1 h heating (ruby) in PBS (PH 9). Wavelength for detection: 254 nm.

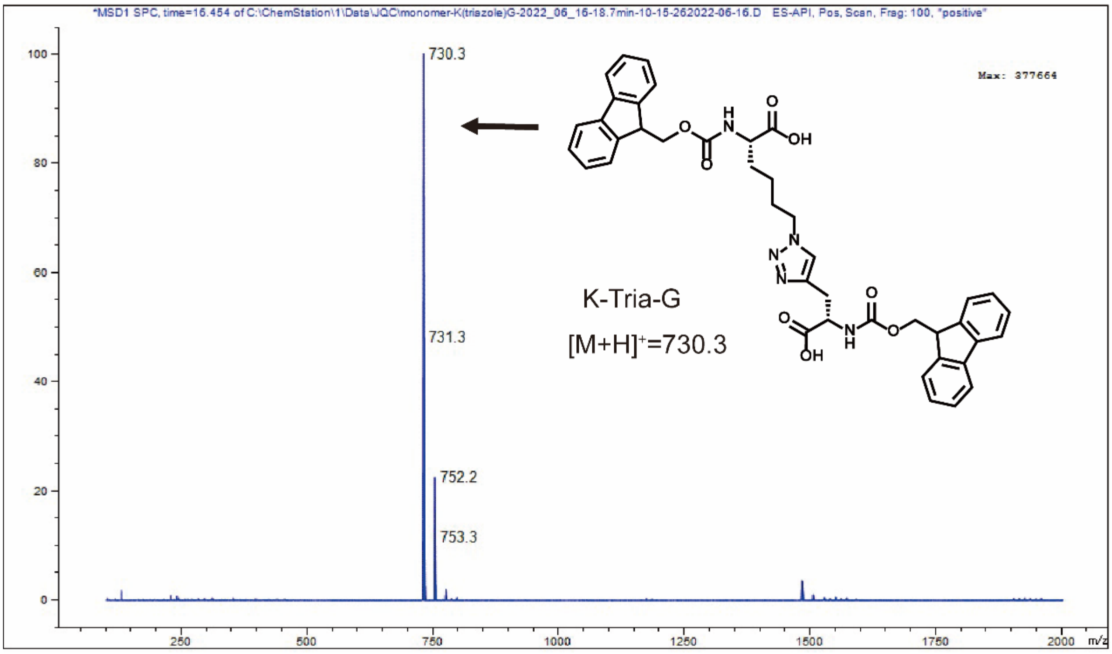


**Supplementary Figure 28.** ESI-MS spectrum of **K-Tria-G**.

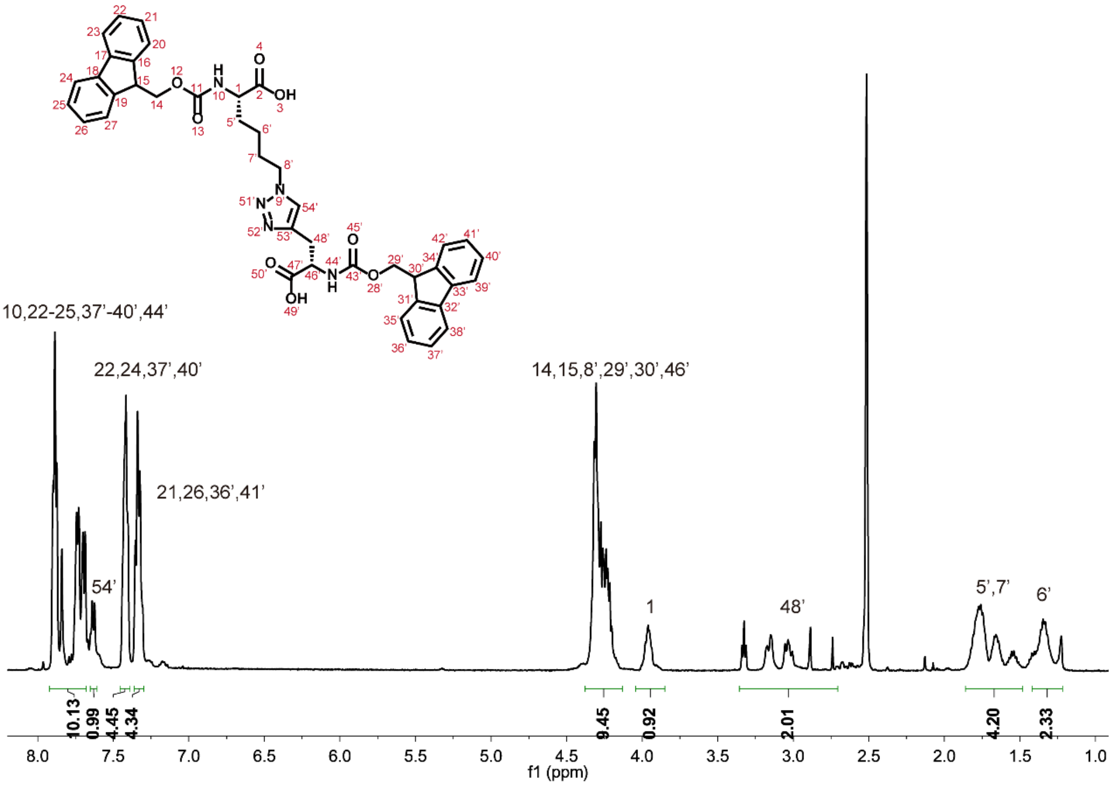


**Supplementary Figure 29.** $^{1}$H NMR (500 MHz, 25 °C) spectrum of **K-Tria-G** in $d_6$-DMSO.

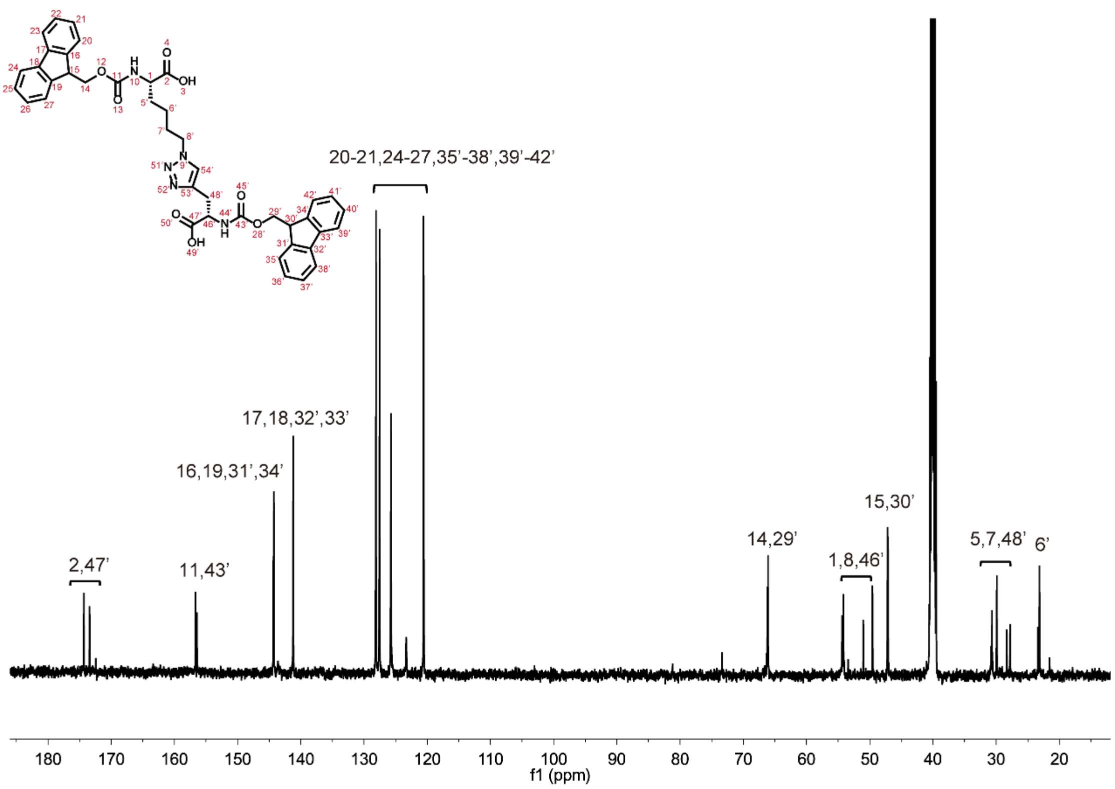


**Supplementary Figure 30.** $^{13}$C NMR (126 MHz, 25 °C) spectrum of **K-Tria-G** in $d_6$-DMSO.

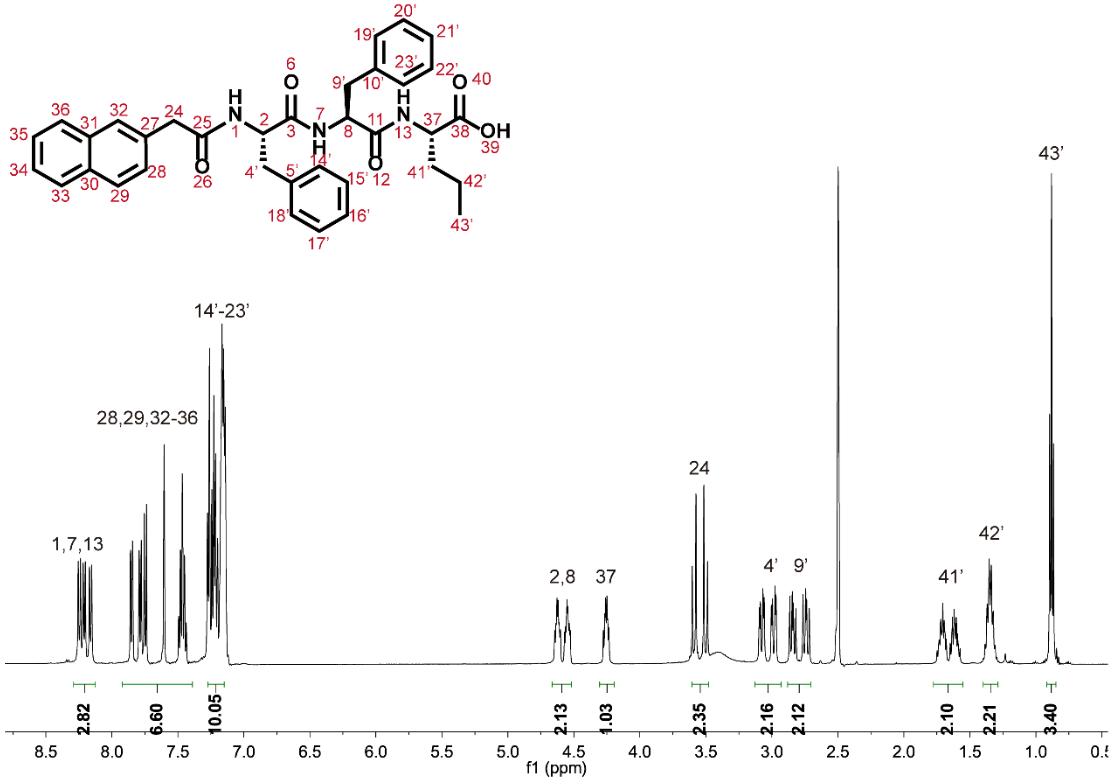


**Supplementary Figure31.** $^{1}$H NMR (500 MHz, 25 °C) spectrum of **Nap-FF-Nva** in $d_6$-DMSO.

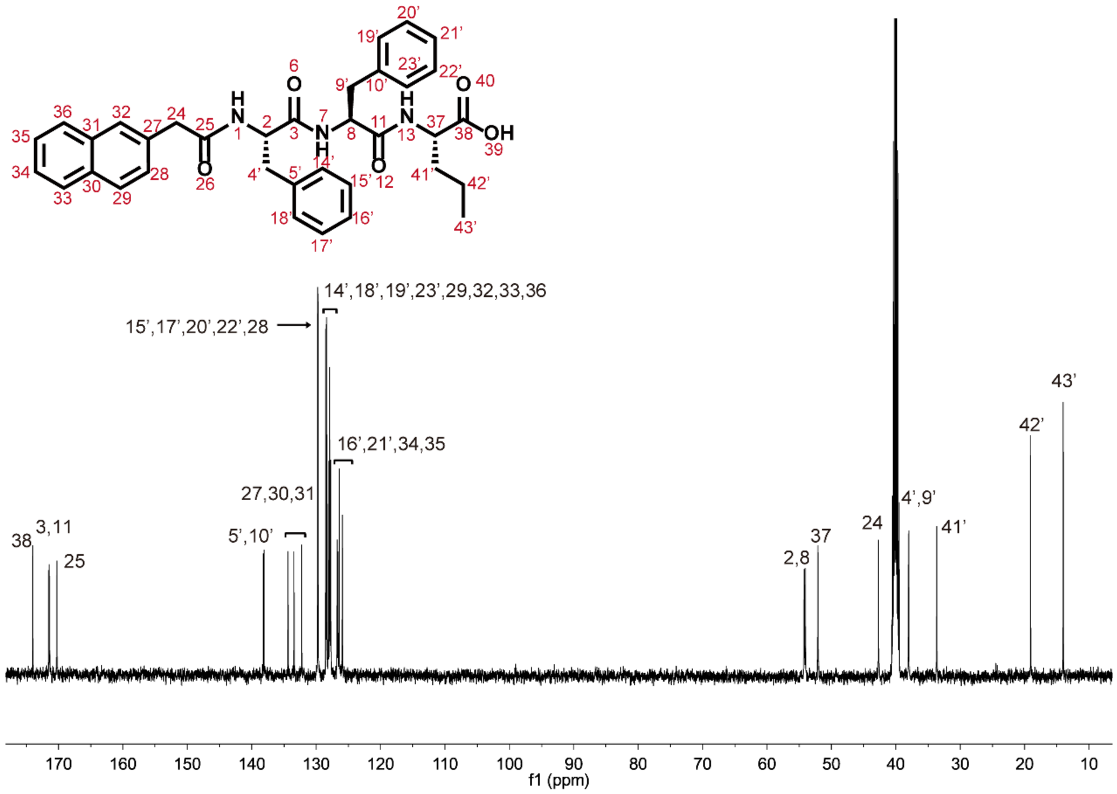


**Supplementary Figure 32.** $^{13}C$ NMR (126 MHz, 25 °C) spectrum of **Nap-FF-Nva** in $d_6$-DMSO.

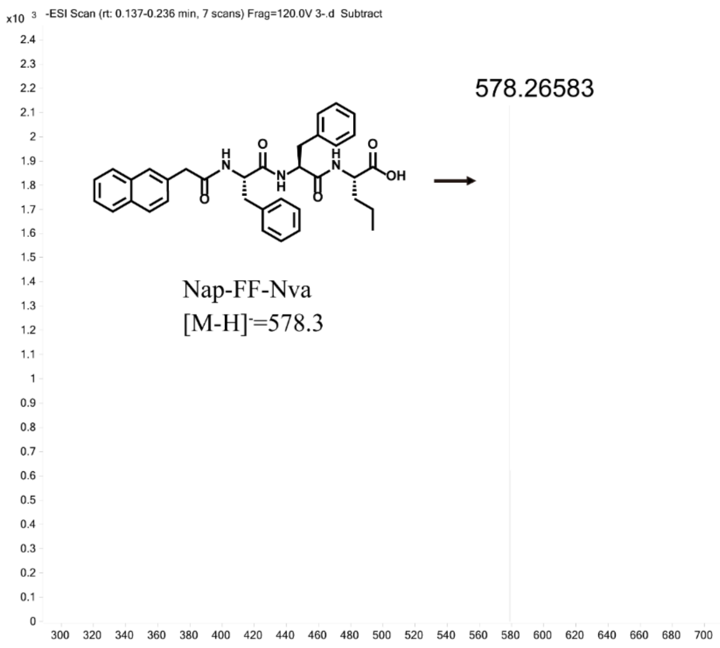


**Supplementary Figure 33.** ESI-MS spectrum of **Nap-FF-Nva**.

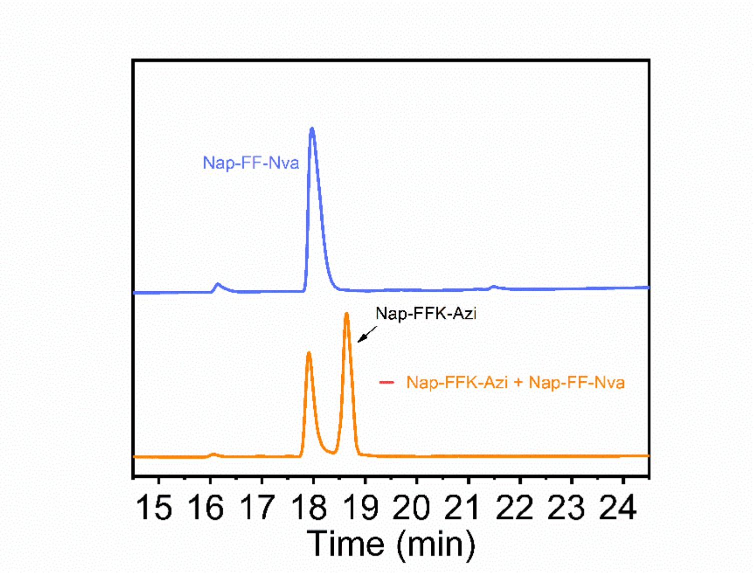


**Supplementary Figure 34.** HPLC traces of 2.5 mM 2.5 mM **Nap-FF-Nva** (wathet), and 2.5 mM **Nap-FFK-Azi** + **Nap-FF-Nva** hydrogel at 4 °C for 7 d (orange). Wavelength for detection: 254 nm.

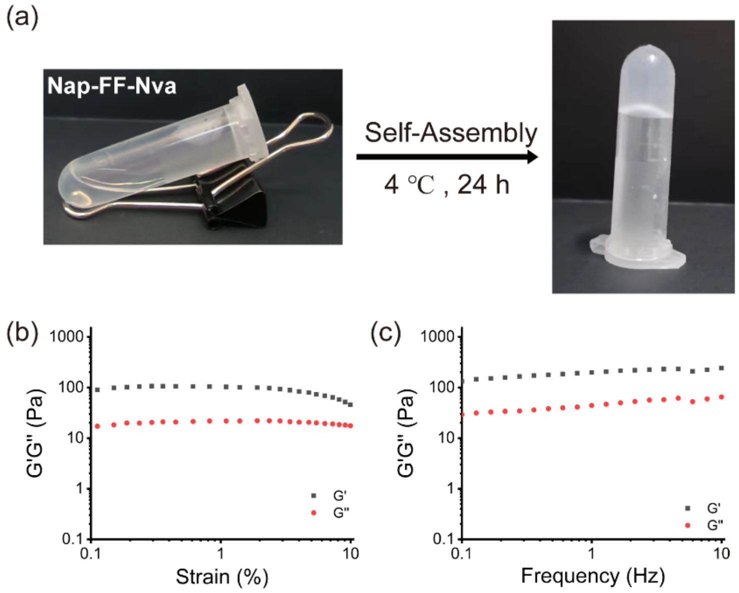


**Supplementary Figure 35.** Photographs of (a) the solution of **Nap-FF-Nva** and its self-assembled hydrogel at 0.32 wt% (2.5 mM) and pH = 9 over 24 h. (b) Strain dependence of the dynamic storage moduli (G′) and the loss moduli (G″) of **Nap-FF-Nva** hydrogel (4 °C, frequency: 1 Hz). (c) Frequency dependence of the dynamic storage moduli (G′) and the loss moduli (G″) of **Nap-FF-Nva** hydrogel. (4 °C, strain: 1.0%).

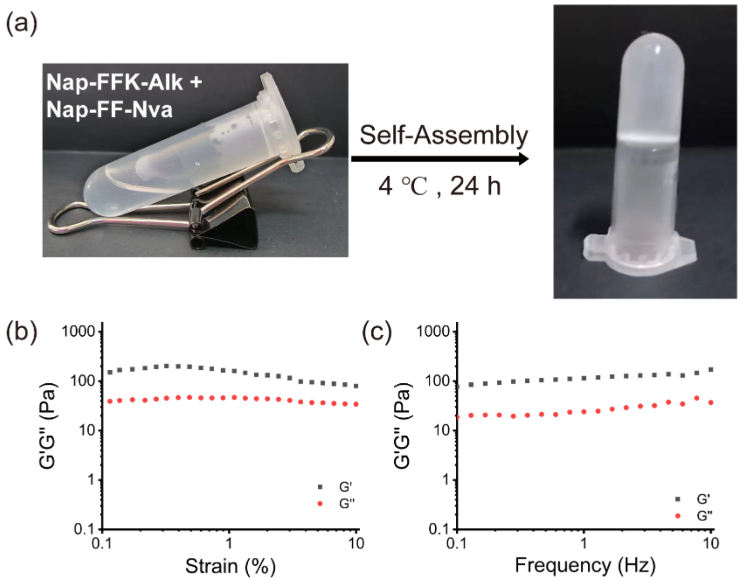


**Supplementary Figure 36.** Photographs of (a) the solution of co-assembly mixture (equal volume **Nap-FFK-Azi** (2.5 mM / 0.15 wt%) and **Nap-FF-Nva** (2.5 mM / 0.32 wt%) and its self-assembled hydrogel at pH = 9 over 24 h. (b) Strain dependence of the dynamic storage moduli (G′) and the loss moduli (G″) of co-assembly mixture (4 °C, frequency: 1 Hz). (c) Frequency dependence of the dynamic storage moduli (G′) and the loss moduli (G″) of co-assembly mixture hydrogel. (4 °C, strain: 1.0%).

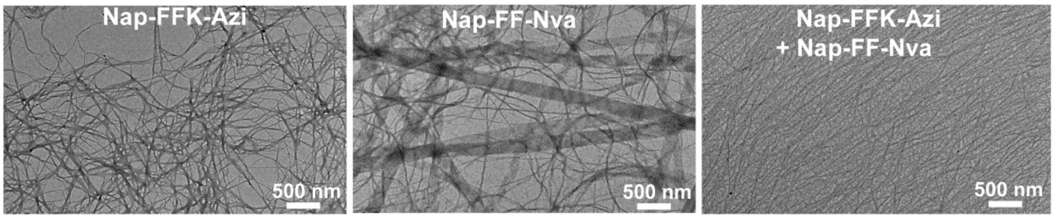


**Supplementary Figure 37.** TEM images of 2.5 mM **Nap-FFK-Azi** hydrogel, 2.5 mM **Nap-FF-Nva** hydrogel, and 2.5 mM **Nap-FFK-Azi + Nap-FF-Nva** hydrogel after aging at 4 °C for 7 d. scale bar = 500 nm.

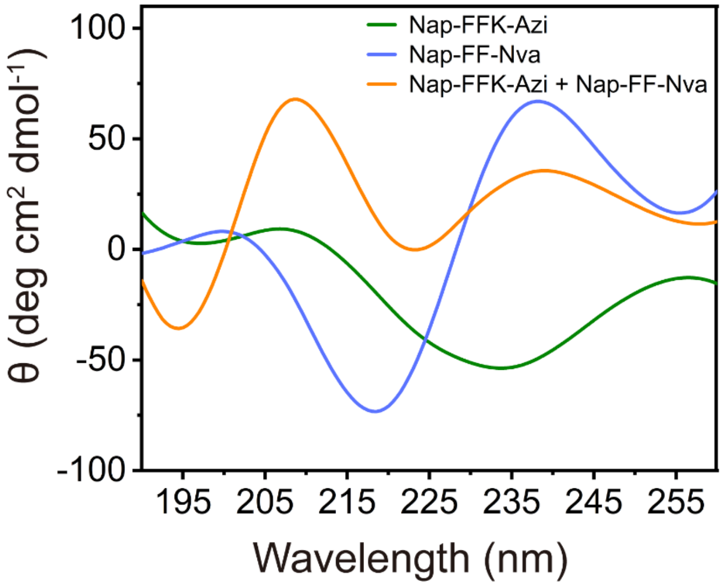


**Supplementary Figure 38.** CD spectra of 2.5 mM **Nap-FFK-Azi** hydrogel, 2.5 mM **Nap-FF-Nva** hydrogel, and 2.5 mM **Nap-FFK-Azi + Nap-FF-Nva** hydrogel after aging at 4 °C for 7 d.

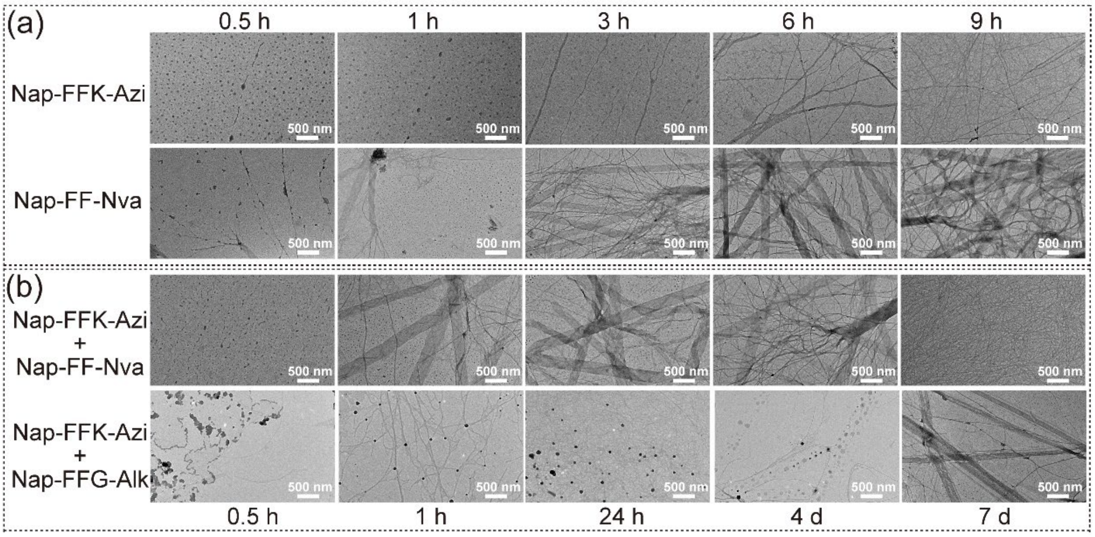


**Supplementary Figure 39. (a)** TEM images of 2.5 mM **Nap-FFK-Azi** hydrogel and 2.5 mM **Nap-FF-Nva** hydrogel at different time points. **(b)** TEM images of 2.5 mM **Nap-FFK-Azi** + **Nap-FF-Nva** hydrogel and **Nap-FFK-Azi** + **Nap-FFG-Alk** hydrogel after aging at 4 °C for 7 d. scale bar = 500 nm.

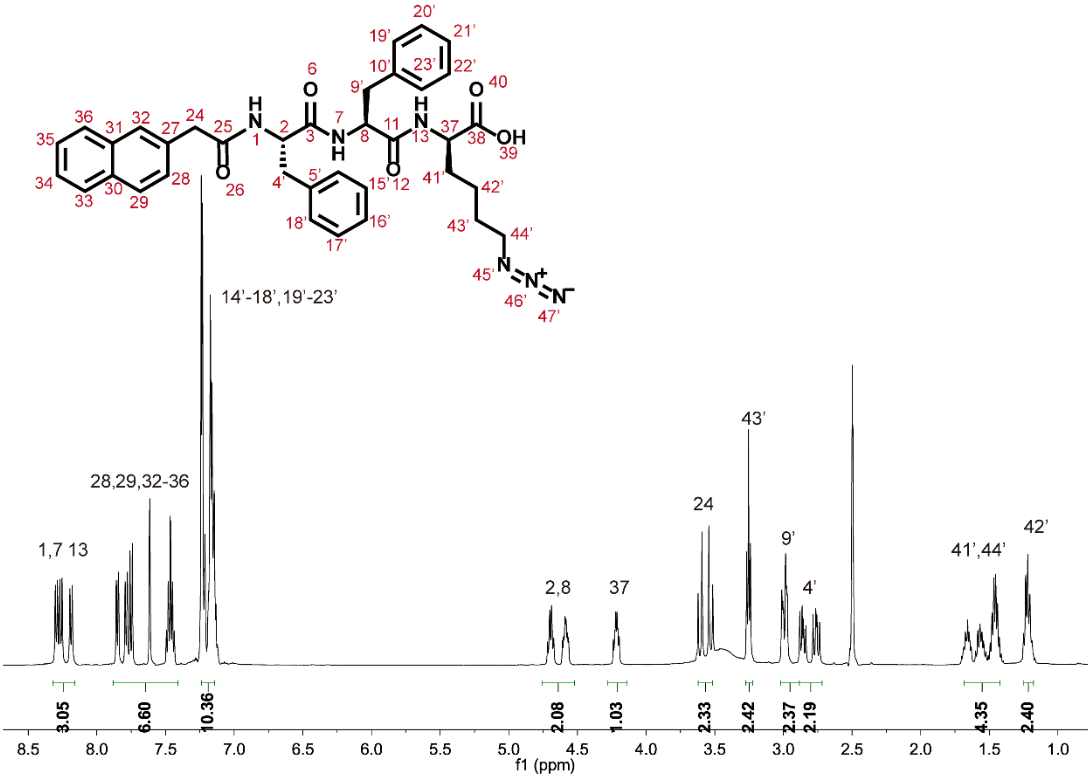


**Supplementary Figure 40.** $^{1}$H NMR (500 MHz, 25 °C) spectrum of **Nap-FFK$_{d}$-Azi** in $d_6$-DMSO.

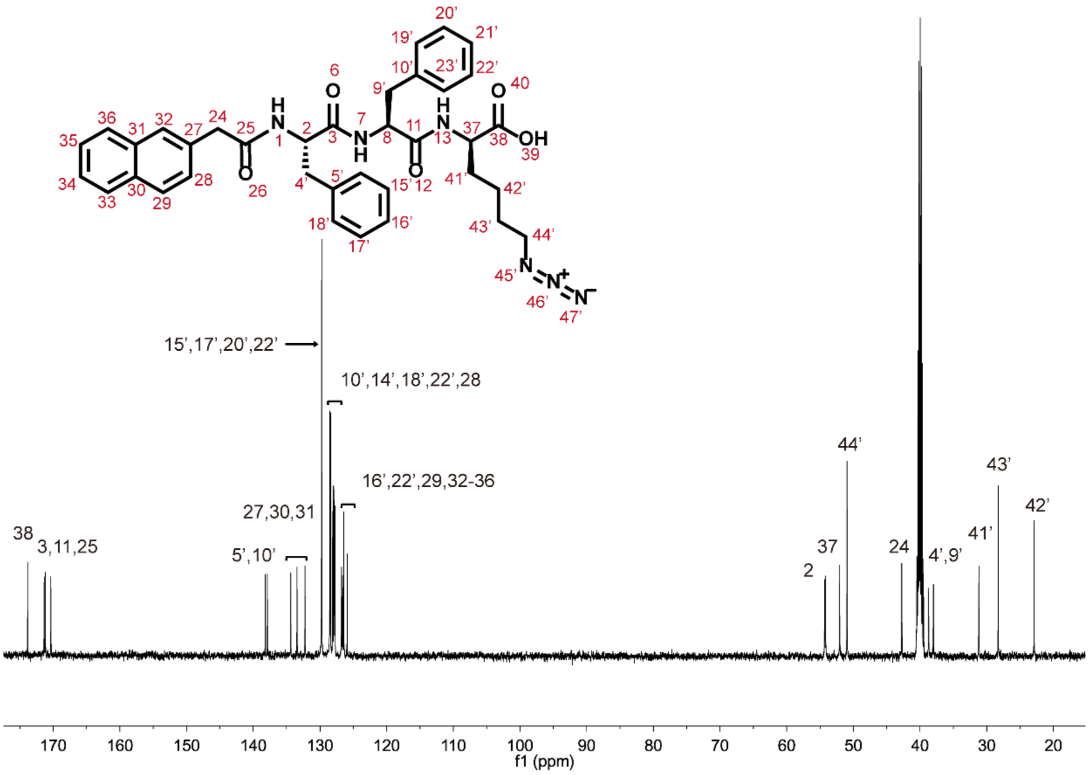


**Supplementary Figure 41.** $^{13}$C NMR (126 MHz, 25 °C) spectrum of **Nap-FFK$_{d}$-Azi** in $d_6$-DMSO.

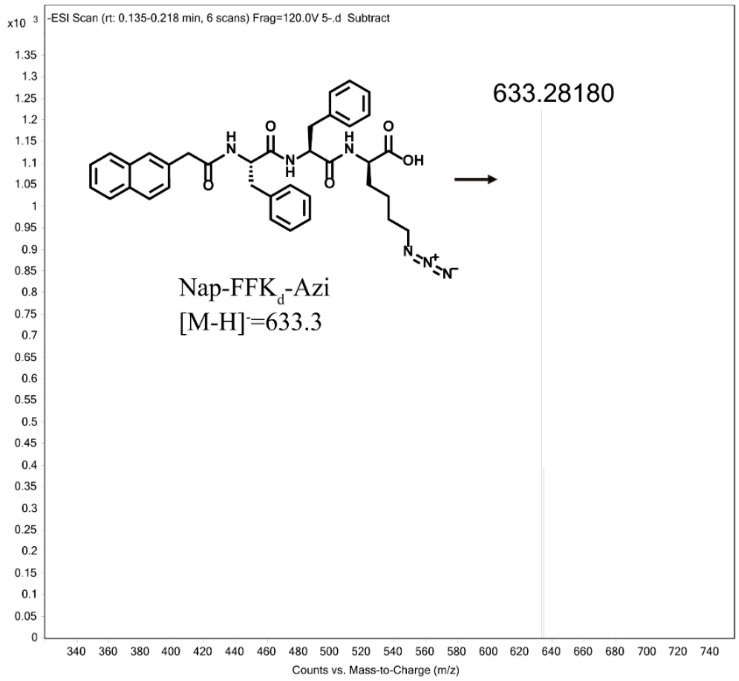


**Supplementary Figure 42.** ESI-MS spectrum of **Nap-FFK$_d$-Azi**.

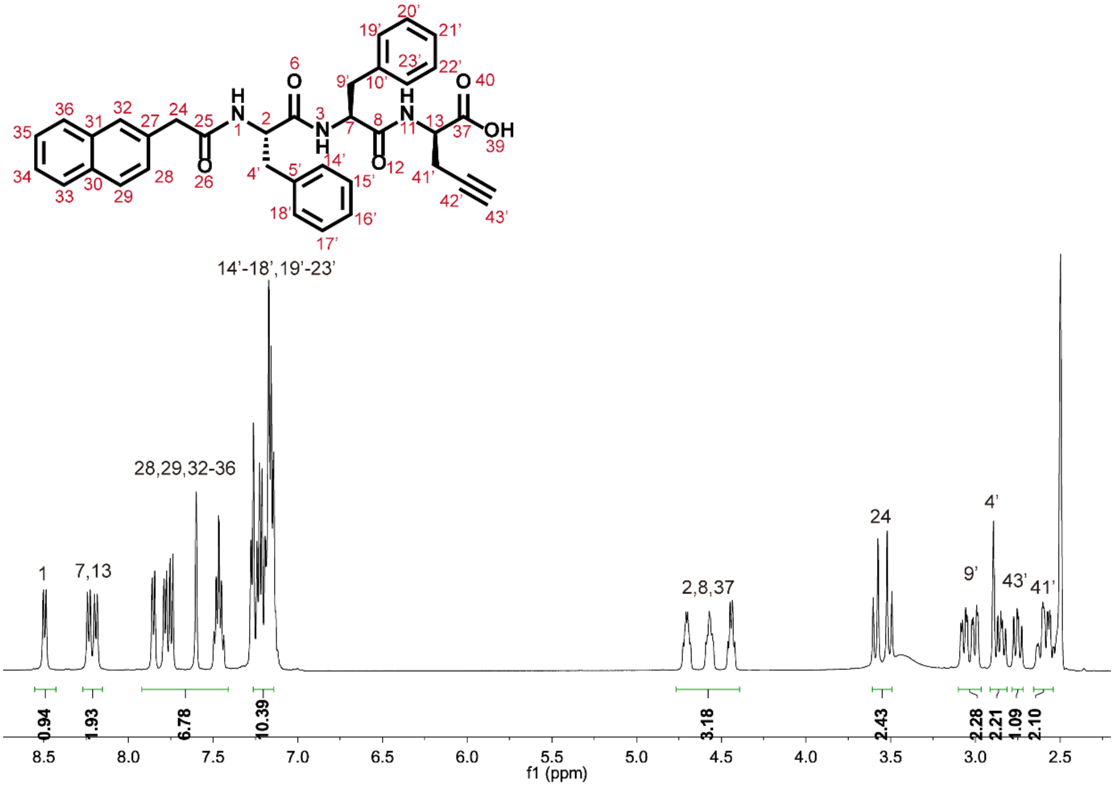


**Supplementary Figure 43.** $^1$H NMR (500 MHz, 25 °C) spectrum of **Nap-FFG$_d$-Alk** in *d$_6$*-DMSO.

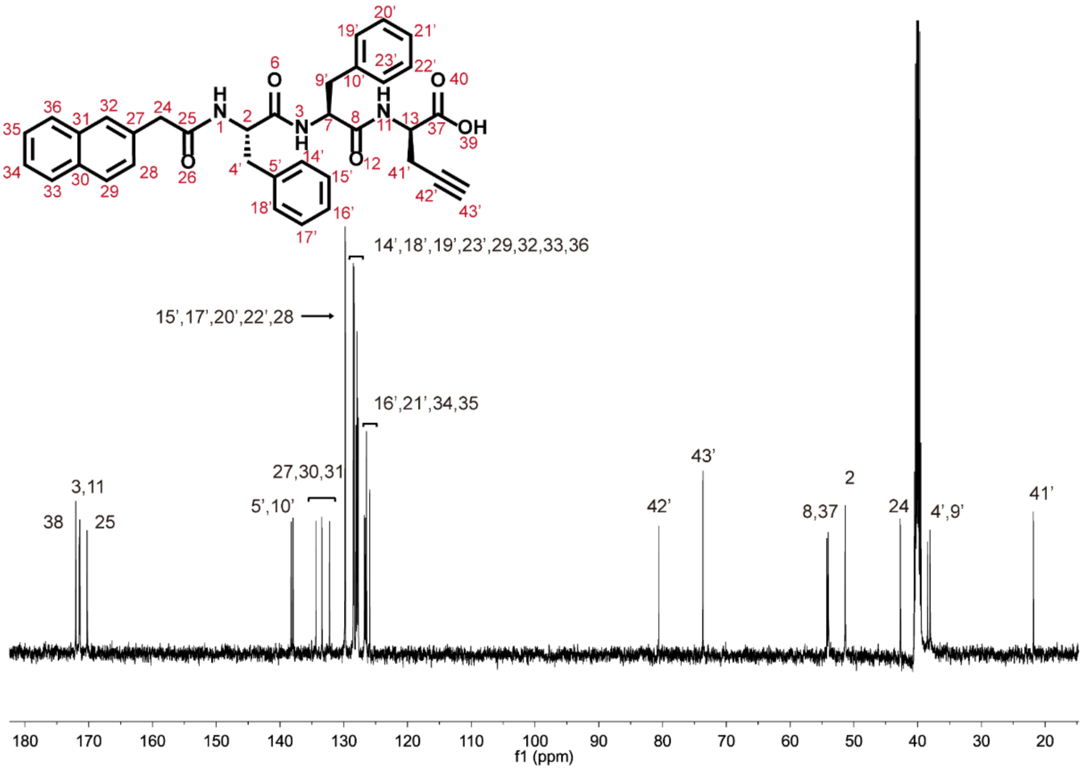


**Supplementary Figure 44.** $^{13}$C NMR (126 MHz, 25 °C) spectrum of **Nap-FFG$_d$-Alk** in $d_6$-DMSO.

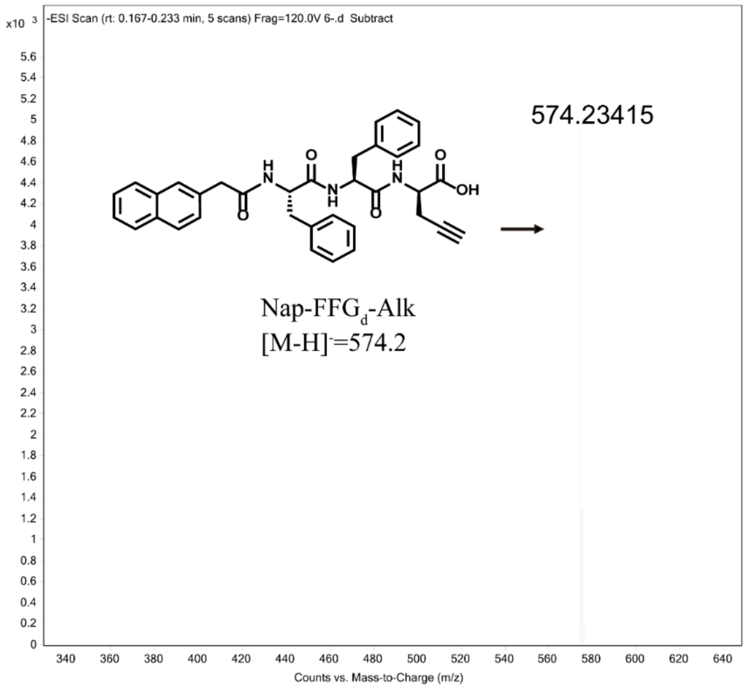


**Supplementary Figure 45.** ESI-MS spectrum of **Nap-FFG$_d$-Alk**.

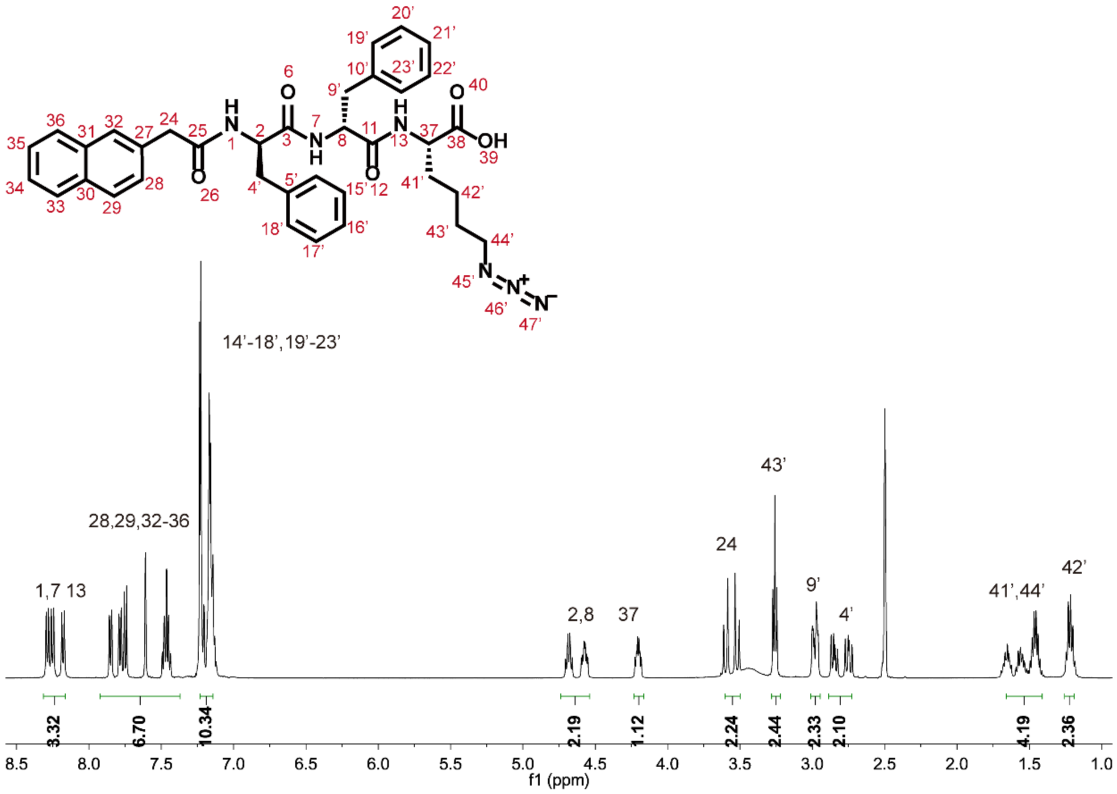


**Supplementary Figure 46.** $^{1}$H NMR (500 MHz, 25 °C) spectrum of **Nap-F$_d$F$_d$K-Azi** in $d_6$-DMSO.

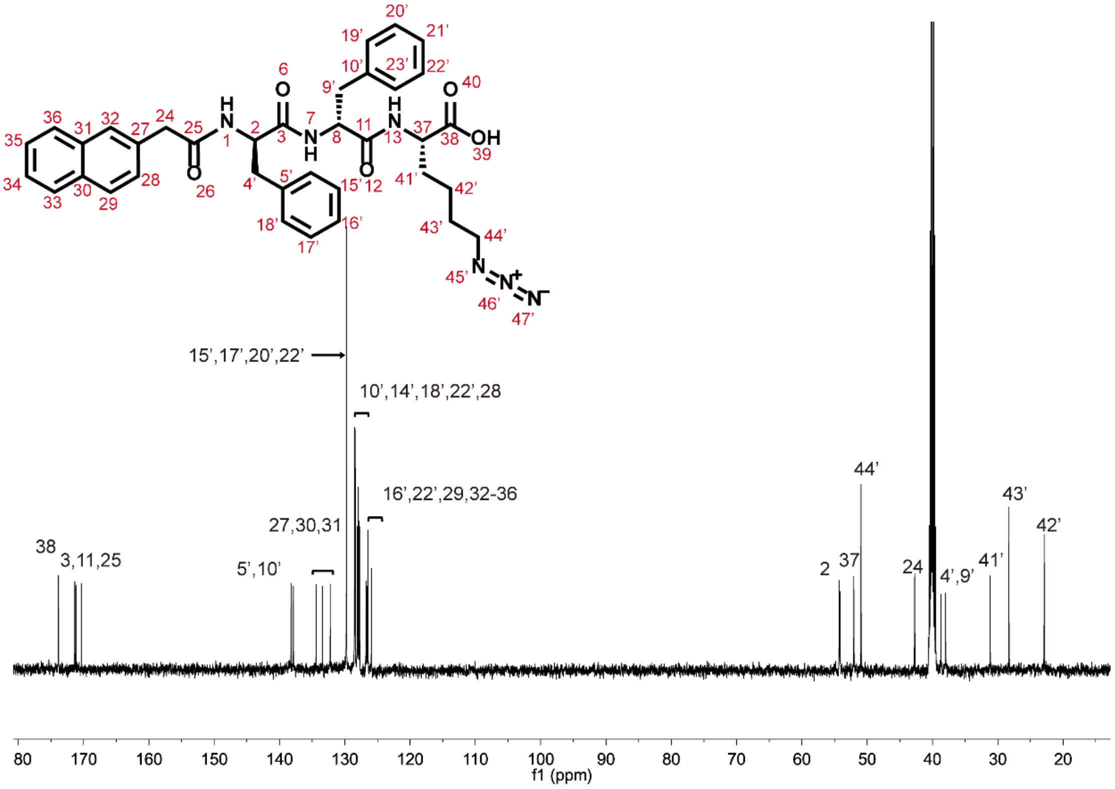


**Supplementary Figure 47.** $^{13}$C NMR (126 MHz, 25 °C) spectrum of **Nap-F$_d$F$_d$K-Azi** in $d_6$-DMSO.

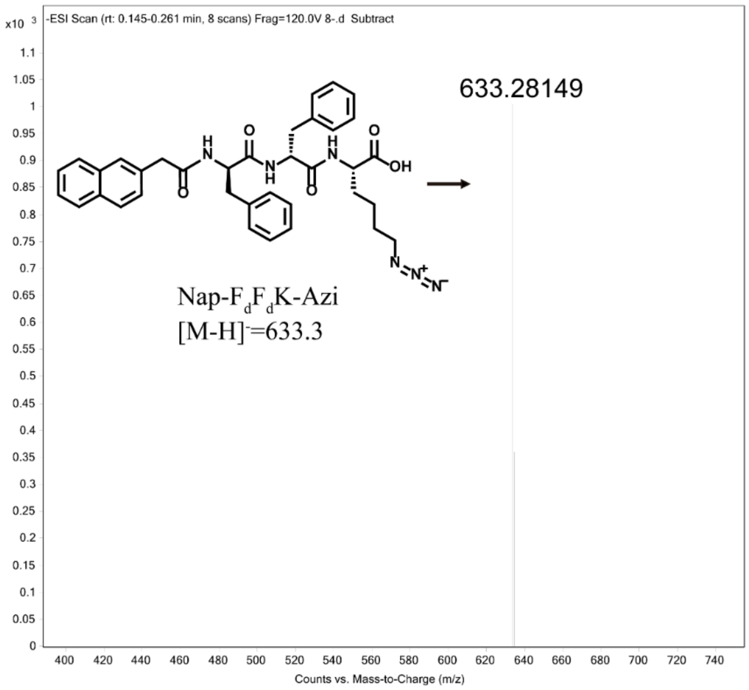


**Supplementary Figure 48.** ESI-MS spectrum of **Nap-F$_d$F$_d$K-Azi**.

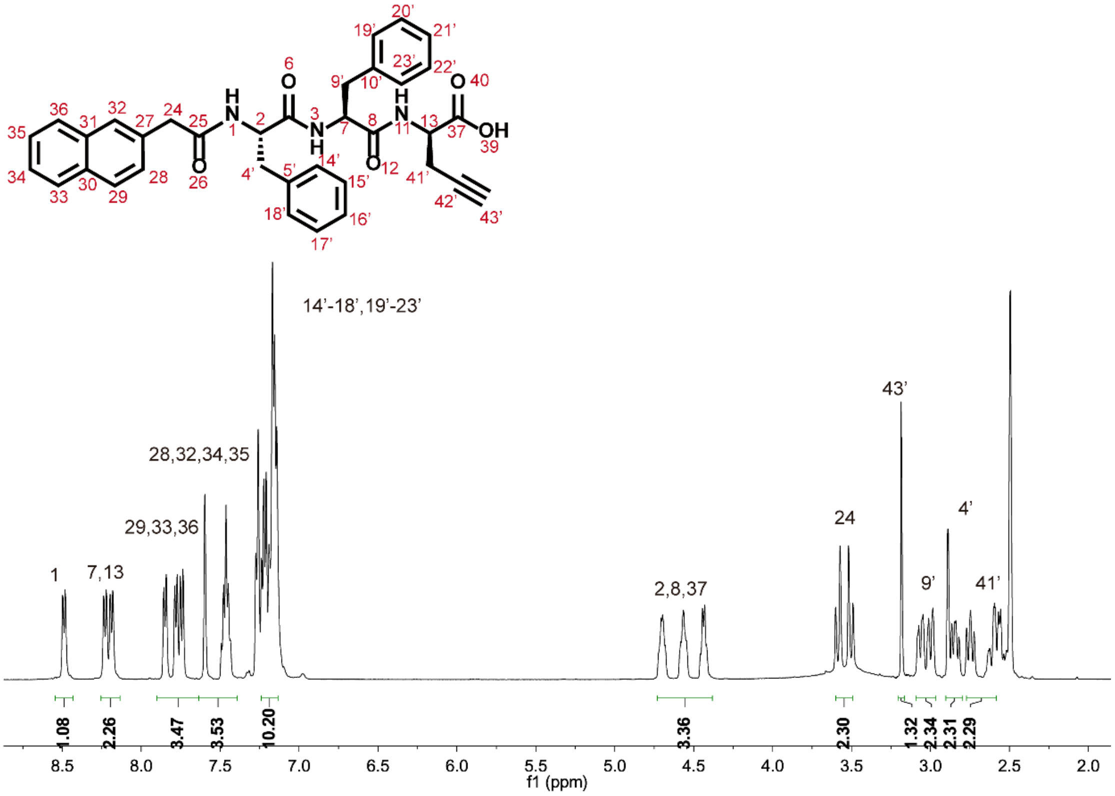


**Supplementary Figure 49.** $^1$H NMR (500 MHz, 25 °C) spectrum of **Nap-F$_d$F$_d$G-Alk** in $d_6$-DMSO.

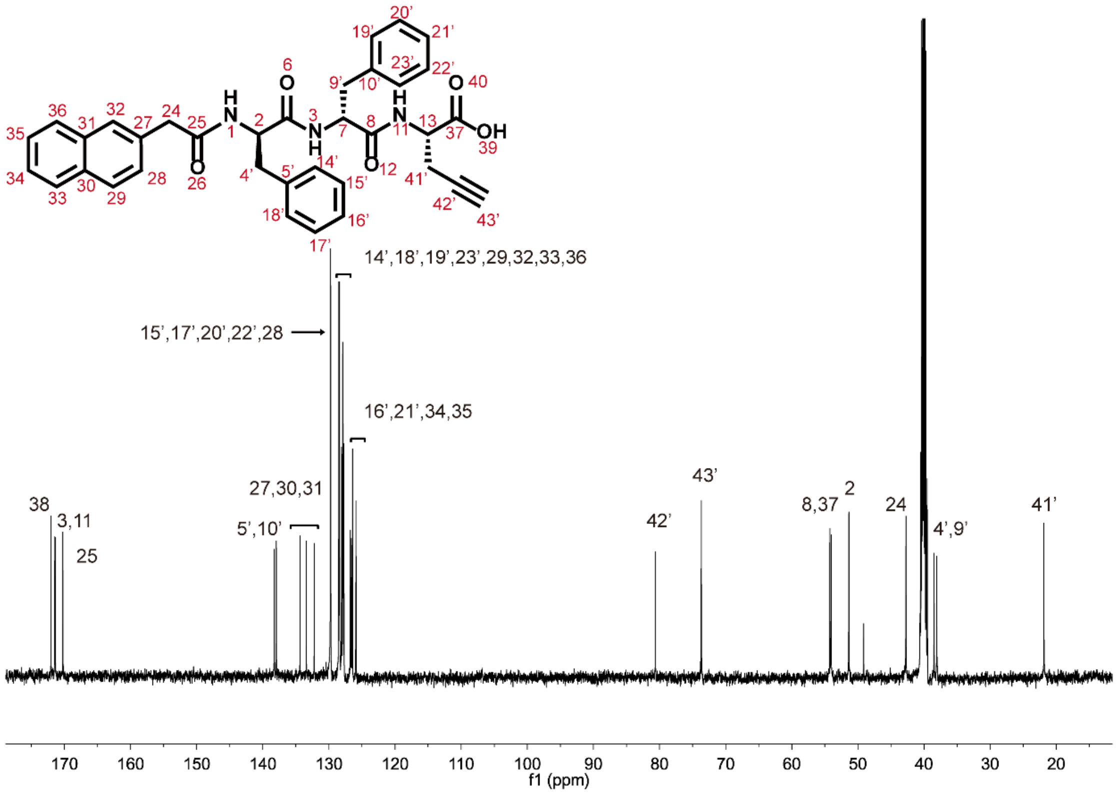


**Supplementary Figure 50.** $^{13}$C NMR (126 MHz, 25 °C) spectrum of **Nap-F$_d$F$_d$G-Alk** in $d_6$-DMSO.

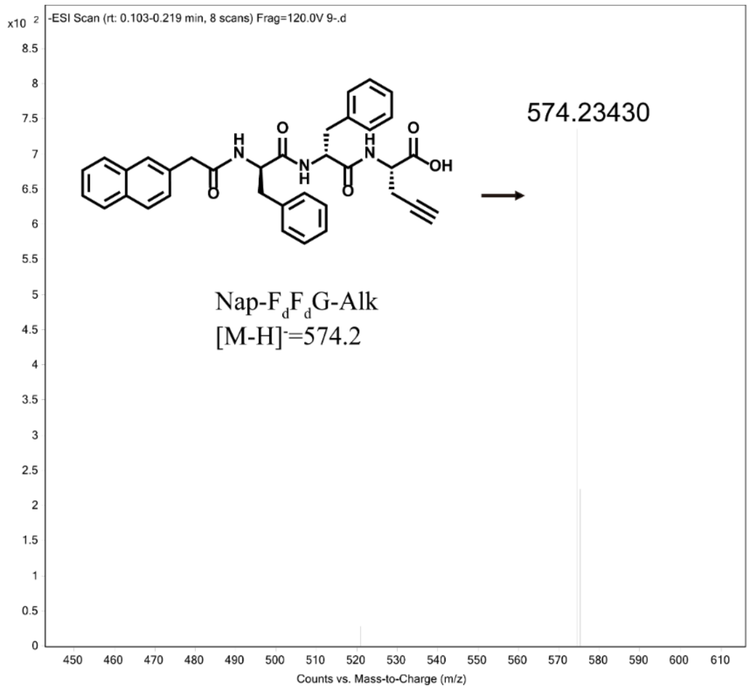


**Supplementary Figure 51.** ESI-MS spectrum of **Nap-F$_d$F$_d$G-Alk**.

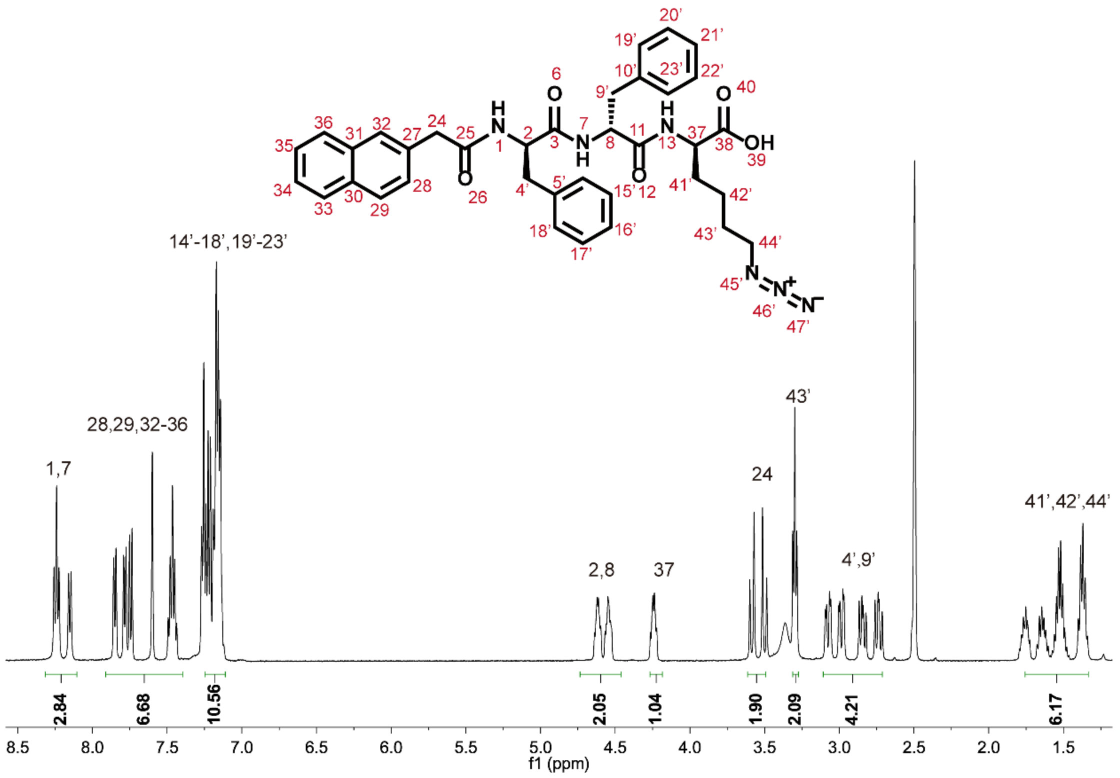


**Supplementary Figure 52.** $^{1}$H NMR (500 MHz, 25 °C) spectrum of **Nap-F$_d$F$_d$K$_d$-Azi** in $d_6$-DMSO.

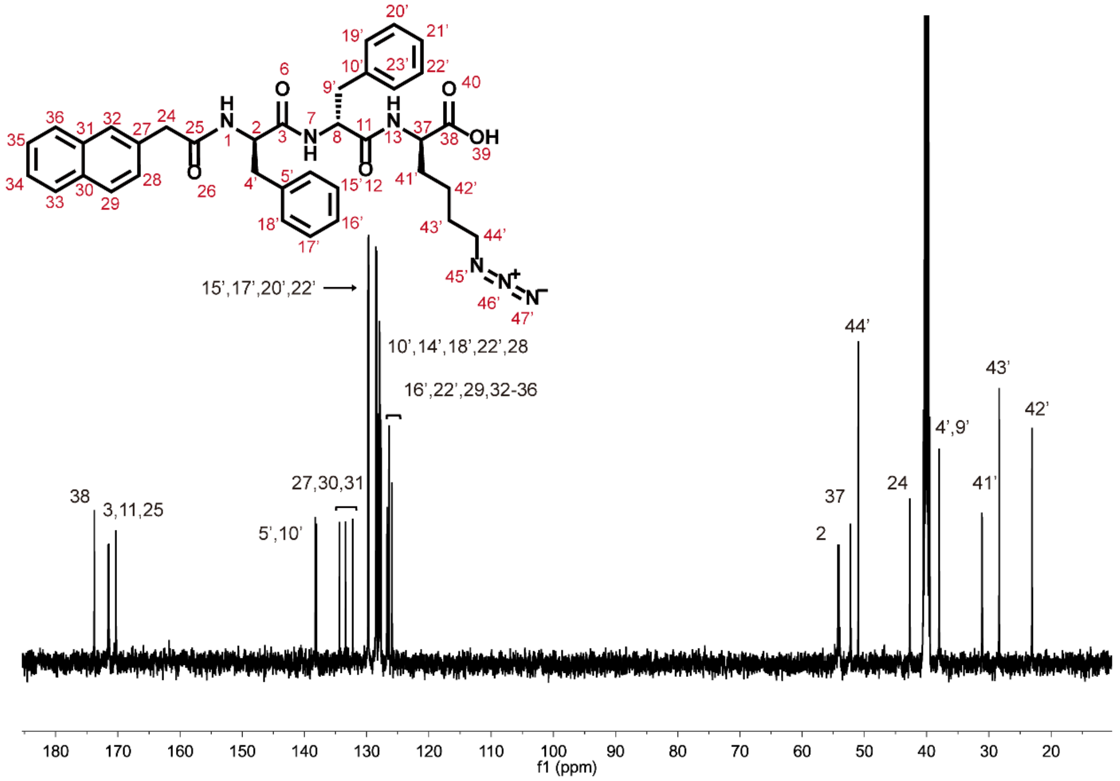


**Supplementary Figure 53.** $^{13}$C NMR (126 MHz, 25 °C) spectrum of **Nap-F$_d$F$_d$K$_d$-Azi** in $d_6$-DMSO.

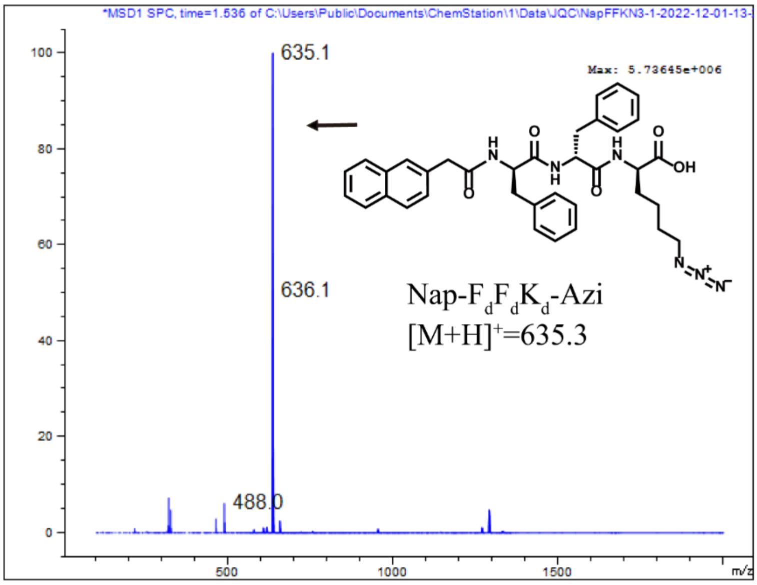


**Supplementary Figure 54.** ESI-MS spectrum of **Nap-F<sub></sub>** 

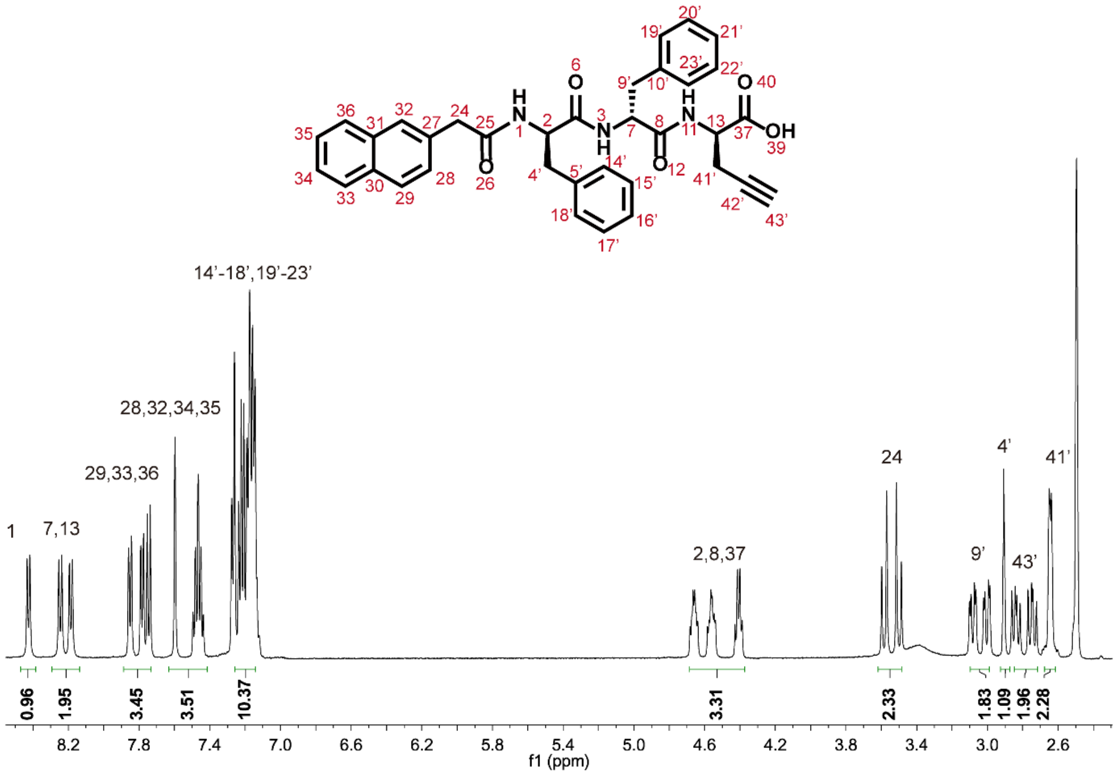


**Supplementary Figure 55.** $^1$H NMR (500 MHz, 25 °C) spectrum of **Nap-$F_dF_dG_d$-Alk** in $d_6$-DMSO.

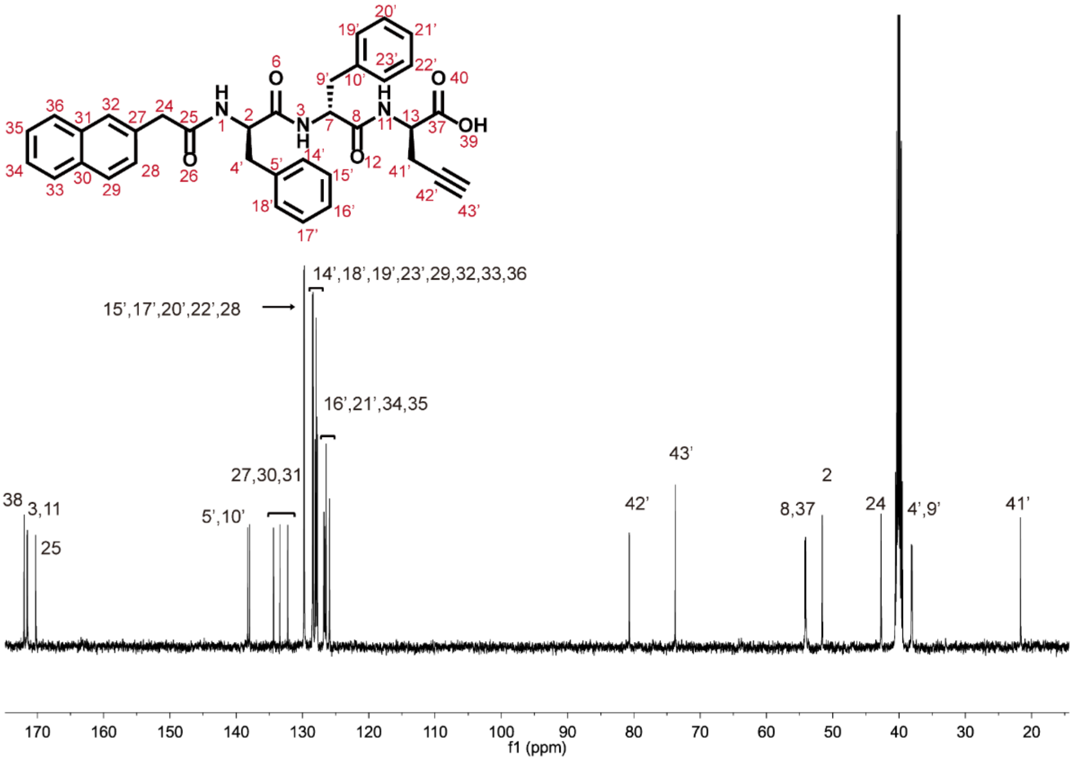


**Supplementary Figure 56.** $^{13}$C NMR (126 MHz, 25 °C) spectrum of **Nap-F$_d$F$_d$G$_d$-Alk** in *d$_6$*-DMSO.

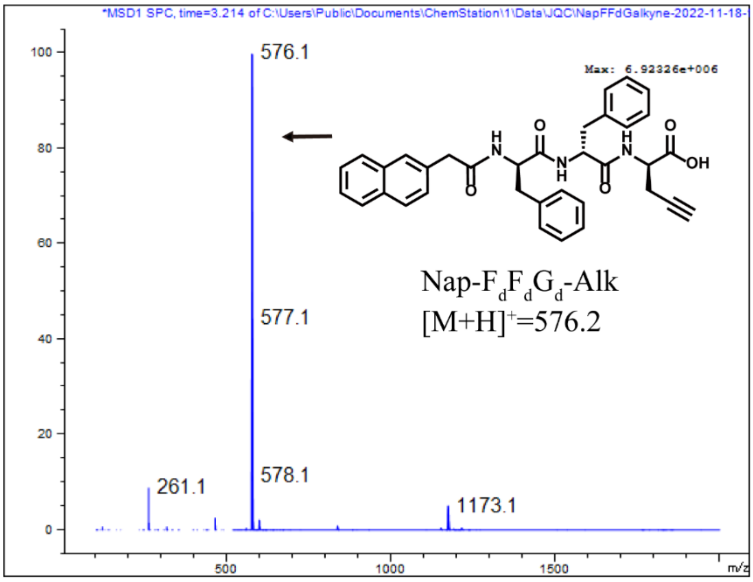


**Supplementary Figure 57.** ESI-MS spectrum of **Nap-F$_d$F$_d$G$_d$-Alk**.

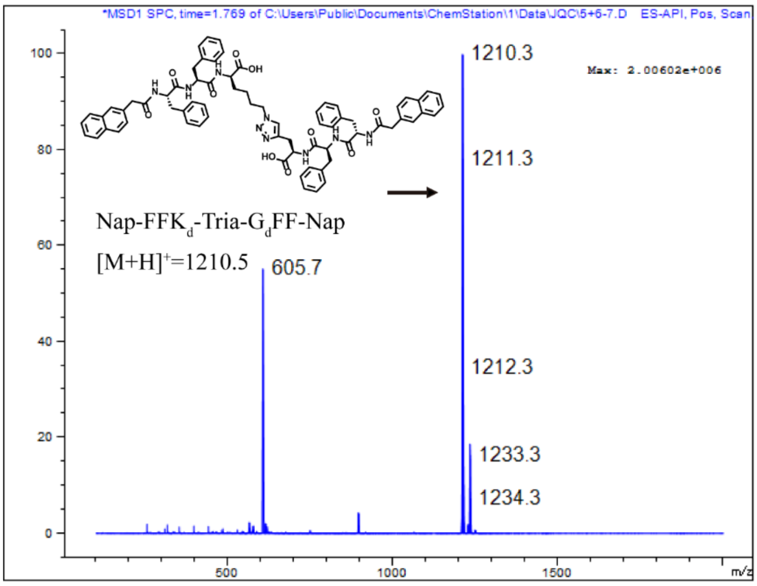


**Supplementary Figure 58.** ESI-MS spectrum of **Nap-FFK$_d$-Tria-G$_d$FF-Nap**.

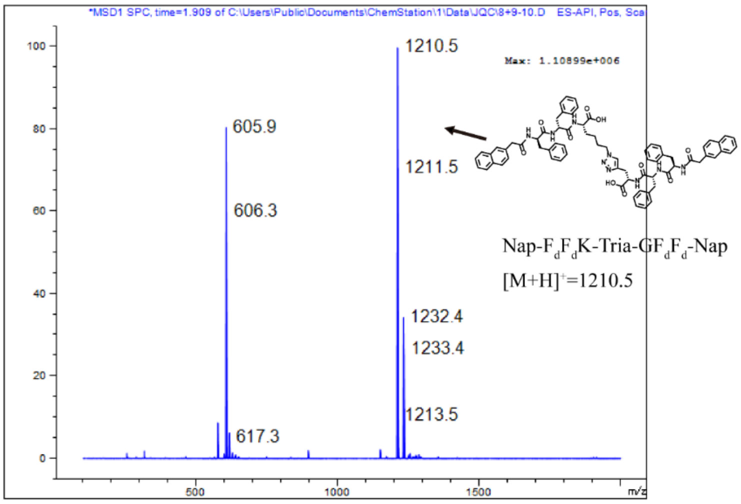


**Supplementary Figure 59.** ESI-MS spectrum of **Nap-F$_d$F$_d$K-Tria-GF$_d$F$_d$-Nap**.

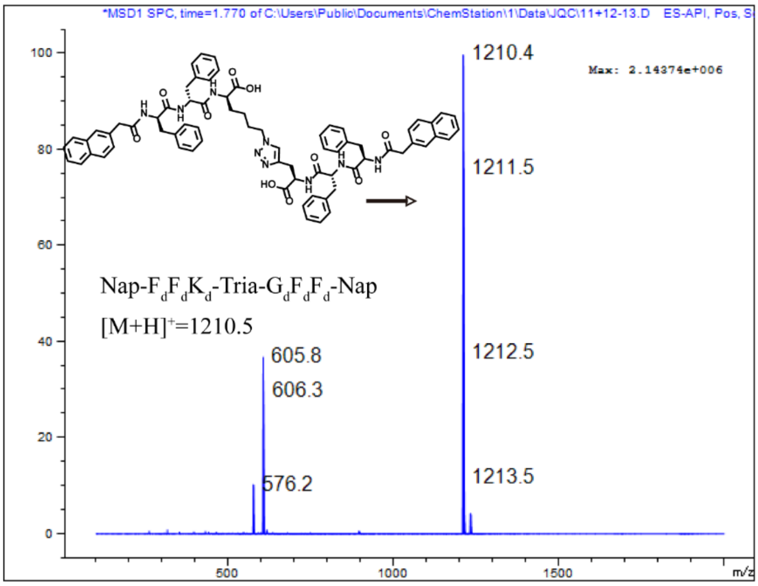


**Supplementary Figure 60.** ESI-MS spectrum of **Nap-F$_d$F$_d$K$_d$-Tria-G$_d$F$_d$F$_d$-Nap**.

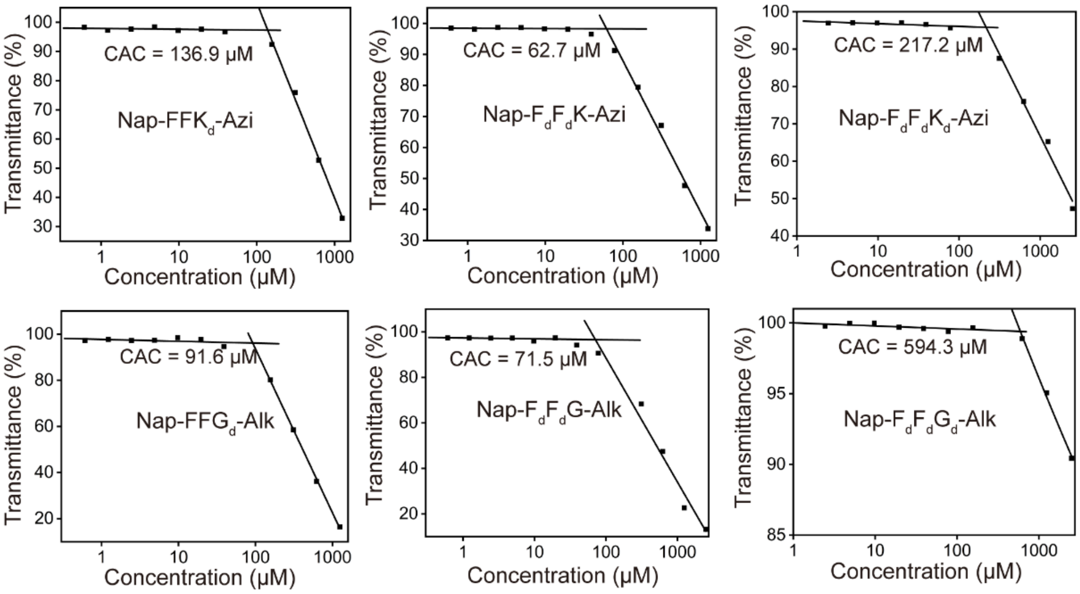


**Supplementary Figure 61.** Critical aggregation concentrations (CACs) of **Nap-FFK$_d$-Azi**, **Nap-FFG$_d$-Alk**, **Nap-F$_d$F$_d$K-Azi**, **Nap-F$_d$F$_d$G-Alk**, **Nap-F$_d$F$_d$K$_d$-Azi**, and **Nap-F$_d$F$_d$G$_d$-Alk**.

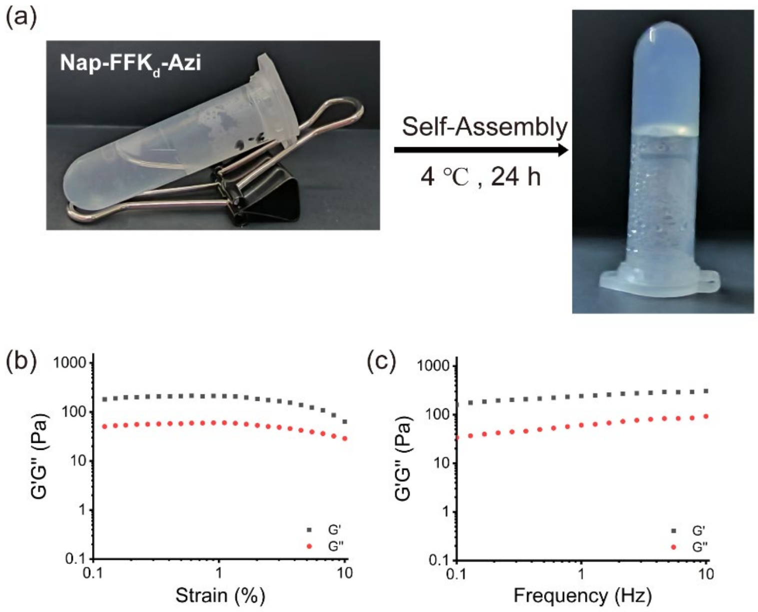


**Supplementary Figure 62.** Photographs of (a) the solution of **Nap-FFK$_d$-Azi** and its self-assembled hydrogel at 0.15 wt% (2.5 mM) and pH = 9 over 24 h. (b) Strain dependence of the dynamic storage moduli (G′) and the loss moduli (G″) of **Nap-FFK$_d$-Azi** hydrogel (4 °C, frequency: 1 Hz). (c) Frequency dependence of the dynamic storage moduli (G′) and the loss moduli (G″) of **Nap-FFK$_d$-Azi** hydrogel. (4 °C, strain: 1.0%).

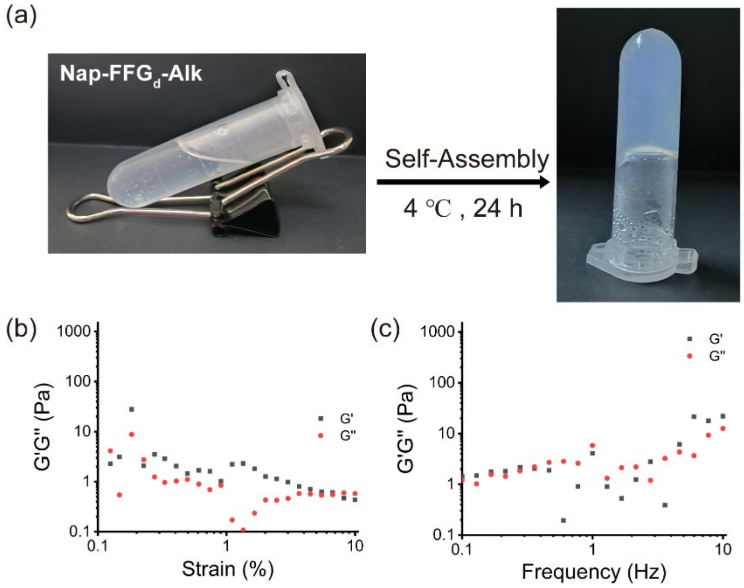


**Supplementary Figure 63.** Photographs of (a) the solution of **Nap-FFG$_d$-Alk** and its self-assembled hydrogel at 0.32 wt% (2.5 mM) and pH = 9 over 24 h. (b) Strain dependence of the dynamic storage moduli (G′) and the loss moduli (G″) of **Nap-FFG$_d$-Alk** hydrogel (4 °C, frequency: 1 Hz). (c) Frequency dependence of the dynamic storage moduli (G′) and the loss moduli (G″) of **Nap-FFG$_d$-Alk** hydrogel. (4 °C, strain: 1.0%).

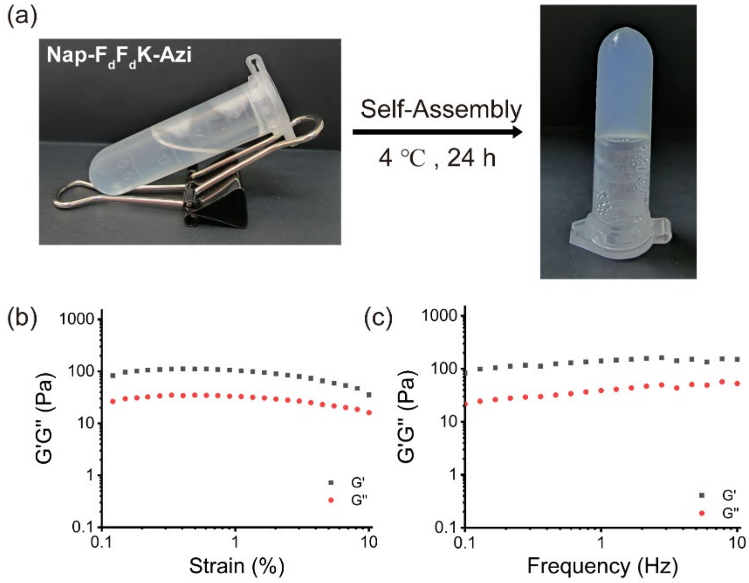


**Supplementary Figure 64.** Photographs of (a) the solution of **Nap-F$_d$F$_d$K-Azi** and its self-assembled hydrogel at 0.15 wt% (2.5 mM) and pH 9 over 24 h. (b) Strain dependence of the dynamic storage moduli (G′) and the loss moduli (G″) of **Nap-F$_d$F$_d$K-Azi** hydrogel (4 ℃, frequency: 1 Hz). (c) Frequency dependence of the dynamic storage moduli (G′) and the loss moduli (G″) of **Nap-F$_d$F$_d$K-Azi** hydrogel. (4 ℃, strain: 1.0 %).

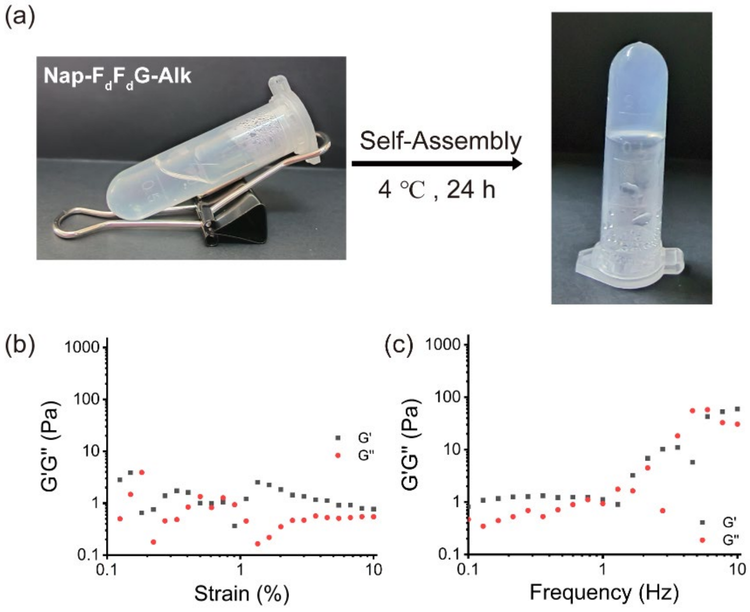


**Supplementary Figure 65.** Photographs of (a) the solution of **Nap-F$_d$F$_d$G-Alk** and its self-assembled hydrogel at 0.32 wt% (2.5 mM) and pH 9 over 24 h. (b) Strain dependence of the dynamic storage moduli (G′) and the loss moduli (G″) of **Nap-F$_d$F$_d$G-Alk** hydrogel (4 ℃, frequency: 1 Hz). (c) Frequency dependence of the dynamic storage moduli (G′) and the loss moduli (G″) of **Nap-F$_d$F$_d$G-Alk** hydrogel. (4 ℃, strain: 1.0%).

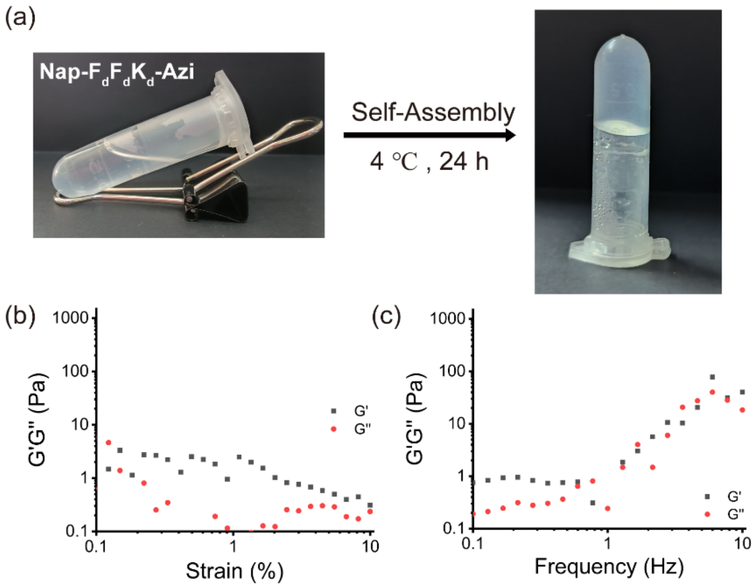


**Supplementary Figure 66.** Photographs of (a) the solution of **Nap-$F_d$$F_d$$K_d$-Azi** and its self-assembled hydrogel at 0.15 wt% (2.5 mM) and pH 9 over 24 h. (b) Strain dependence of the dynamic storage moduli (G′) and the loss moduli (G″) of **Nap-$F_d$$F_d$$K_d$-Azi** hydrogel (4 °C, frequency: 1 Hz). (c) Frequency dependence of the dynamic storage moduli (G′) and the loss moduli (G″) of **Nap-$F_d$$F_d$$K_d$-Azi** hydrogel. (4 °C, strain: 1.0%).

**Supplementary Table 1.** HPLC condition for all the corresponding experiments.

| Time (min) | Flow (mL/min) | $H_2O$ % (0.1% TFA) | $CH_3CN$ % (0.1% TFA) |
|---|---|---|---|
| 0 | 3.0 | 70 | 30 |
| 5 | 3.0 | 70 | 30 |
| 25 | 3.0 | 0 | 100 |
| 30 | 3.0 | 70 | 30 |

**Supplementary Table 2.** Quantitative analyses of the HPLC peaks on the trace in Figure 1b.

| Name | Start (min) | End (min) | Retention (mins) | Height (CPM) | Area (CPM) | %ROI (%) |
|---|---|---|---|---|---|---|
| Region 1 | 15.879 | 16.532 | 16.087 | 773.634 | 9,195.619 | 12.847 |
| Region 2 | 17.052 | 17.748 | 17.264 | 2870.839 | 36441.934 | 50.912 |
| Region 3 | 20.128 | 20.638 | 20.344 | 2898.189 | 25939.667 | 36.241 |
| 3 Peaks | | | | | 71,577.22 | 100 |

**Supplementary Table 3.** Quantitative analyses of the HPLC peaks on the trace in Figure 2a.

| Name | Start (min) | End (min) | Retention (mins) | Height (CPM) | Area (CPM) | %ROI (%) |
|---|---|---|---|---|---|---|
| Region 1 | 17.001 | 17.424 | 17.296 | 118.353 | 1060.724 | 44.54 |
| Region 2 | 17.424 | 17.695 | 17.531 | 28.764 | 197.702 | 4.281 |
| Region 3 | 18.412 | 18.774 | 18.666 | 91.842 | 628.544 | 26.479 |
| Region 4 | 18.774 | 19.231 | 18.850 | 34.453 | 258.038 | 10.010 |
| Region 5 | 20.106 | 20.536 | 20.303 | 53.354 | 378.647 | 14.690 |
| 5 Peaks | | | | | 2523.655 | 100 |
